# Targeted resequencing of 358 candidate genes for autism spectrum disorder in a Chinese cohort reveals diagnostic potential and genotype–phenotype correlations

**DOI:** 10.1002/humu.23724

**Published:** 2019-04-29

**Authors:** Wei‐Zhen Zhou, Jie Zhang, Ziyi Li, Xiaojing Lin, Jiarui Li, Sheng Wang, Changhong Yang, Qixi Wu, Adam Yongxin Ye, Meng Wang, Dandan Wang, Tad Zhengzhang Pu, Yu‐Yu Wu, Liping Wei

**Affiliations:** ^1^ Center for Bioinformatics, State Key Laboratory of Protein and Plant Gene Research, School of Life Sciences Peking University Beijing China; ^2^ State Key Laboratory of Cardiovascular Disease, Beijing Key Laboratory for Molecular Diagnostics of Cardiovascular Diseases, Diagnostic Laboratory Service, Fuwai Hospital, National Center for Cardiovascular Diseases Chinese Academy of Medical Sciences and Peking Union Medical College Beijing China; ^3^ National Institute of Biological Sciences Beijing China; ^4^ College of Biological Sciences China Agricultural University Beijing China; ^5^ College of Life Sciences Beijing Normal University Beijing China; ^6^ School of Life Sciences Peking University Beijing China; ^7^ Peking‐Tsinghua Center for Life Sciences Beijing China; ^8^ Academy for Advanced Interdisciplinary Studies Peking University Beijing China; ^9^ Shanghai United Family Hospital and Clinics Shanghai China; ^10^ Yuning Psychiatry Clinic Taipei Taiwan

**Keywords:** autism, Chinese, genetic testing, targeted resequencing

## Abstract

Autism spectrum disorder (ASD) is a childhood neuropsychiatric disorder with a complex genetic architecture. The diagnostic potential of a targeted panel of ASD genes has only been evaluated in small cohorts to date and is especially understudied in the Chinese population. Here**,** we designed a capture panel with 358 genes (111 syndromic and 247 nonsyndromic) for ASD and sequenced a Chinese cohort of 539 cases evaluated with the Autism Diagnostic Interview‐Revised (ADI‐R) and the Autism Diagnostic Observation Schedule (ADOS) as well as 512 controls. ASD cases were found to carry significantly more ultra‐rare functional variants than controls. A subset of 78 syndromic and 54 nonsyndromic genes was the most significantly associated and should be given high priority in the future screening of ASD patients. Pathogenic and likely pathogenic variants were detected in 9.5% of cases. Variants in *SHANK3* and *SHANK2* were the most frequent, especially in females, and occurred in 1.2% of cases. Duplications of 15q11–13 were detected in 0.8% of cases. Variants in *CNTNAP2* and *MEF2C* were correlated with epilepsy/tics in cases. Our findings reveal the diagnostic potential of ASD genetic panel testing and new insights regarding the variant spectrum. Genotype–phenotype correlations may facilitate the diagnosis and management of ASD.

## 1 INTRODUCTION

Autism spectrum disorder (ASD) is a neurodevelopmental disorder with symptoms of social interaction and communication deficits, repetitive behavior, and restricted interest (American Psychiatric Association, 2013). ASD displays a high heritability with a complex genetic architecture (de la Torre‐Ubieta, Won, Stein, & Geschwind, 2005). Syndromic ASD is typically comorbid with a single‐gene Mendelian disorder. Each syndromic disorder is typically found in <1% of ASD patients, and altogether, they contribute to about 5–10% of the total ASD population (Betancur, 2011). The genetic causes of nonsyndromic ASD are more complex and often difficult to pinpoint in a patient; among them, *de novo* (DN) loss‐of‐function (LoF) variants appear to have larger effect sizes (Iossifov et al., 2014; T. Wang et al., 2016).

At present, Sanger sequencing and chromosomal microarrays (CMAs) are often recommended as genetic testing approaches for most common ASD syndromes and for large chromosomal abnormalities, respectively (Carter & Scherer, 2010). Next‐generation sequencing has the potential to detect pathogenic variants on a whole genome or whole exome scale, especially in patients without clinical evidence of associated syndromes. Multigene panels have already been established as cost‐effective technologies for clinical diagnoses of many diseases, such as cancer, cardiovascular diseases, and metabolic disorders (Rehm, 2013). Especially for disorders with high genetic heterogeneity like ASD, targeted resequencing could increase the molecular diagnostic sensitivity and significantly reduce cost by focusing on genes with available evidence associated with ASD.

Alvarez‐Mora et al. (2016) designed an ASD panel comprised of 44 candidate genes and conducted a pilot study in 50 patients. However, they reported a low diagnostic yield, possibly because of the small size of the panel and cohort. There is a need for a more comprehensive panel and a larger ASD cohort to evaluate the diagnostic potential and study the variant spectrum and genotype–phenotype correlations.

Most known ASD genes have been identified in Caucasian populations, and their association with ASD in Chinese populations has not been established beyond specific candidate gene association studies (de la Torre‐Ubieta et al., 2005). The correlations between genetic variants and clinical features also remain unexplored in Chinese patients. These gaps may hinder the development of genetic testing and management of ASD in the Chinese population. Here, we developed a comprehensive gene panel covering 358 ASD genes and sequenced an extensively phenotyped Chinese cohort (539 cases and 512 controls). We sought to test the panel, ascertain the variant spectrum for potential future genetic diagnoses in Chinese patients, and understand genotype–phenotype correlations.

## 2 MATERIALS AND METHODS

### 2.1 Editorial policies and ethical considerations

This study was approved by the Peking University Institutional Review Board (IRB00001052‐11043 and IRB00001052‐14055). All subjects or their legal guardians completed written informed consent.

### 2.2 Subjects

We recruited ASD families from training centers in Beijing and Tsingdao, China. All patients had a clinical diagnosis of ASD and underwent our assessments. In particular, they were evaluated with the current “gold standard” diagnostic tools, the Autism Diagnostic Interview‐Revised (ADI‐R; Lord, Rutter, & Le Couteur, 1994) and the Autism Diagnostic Observation Schedule (ADOS; Lord et al., 1989) by assessors with certified reliability. We also conducted assessments and questionnaires regarding the patients’ developmental, medical, and family histories. A full list of the assessment tools used in this study is available in Table S1. To be included in our study as a case, the patient was required to satisfy the same ADI‐R criteria for ASD as used by the Simons Simplex Collection (SSC; Fischbach & Lord, 2010). Namely, a child was classified as having ASD if he/she met the ADI‐R cutoffs in the Social and Communication domains, scored within two points of the cutoffs in either the Social or Communication domain, or scored within one point in both domains.

We collected control samples from blood donors at blood donation stations in Beijing, China. All participants, aged 18–55 years old, were born before 2000. Considering that the first Chinese autism cases were reported in 1982 (Tao, 1982), and in China, there were very few hospitals that could diagnose ASD in the 1990s (Zhou et al., 2014), these donors likely had little chance of being diagnosed if they suffered from ASD. To reduce the influence of unrecognized ASD in the donors, we adopted the Adult Autism Spectrum Quotient (AQ; Baron‐Cohen, Wheelwright, Skinner, Martin, & Clubley, 2001) to screen for ASD in the donors. The recommended cutoff (an AQ of 32) was used (Baron‐Cohen et al., 2001). Meanwhile, we also designed the questionnaire to collect other information, including personal and family medical history, as well as pregnancy history, to exclude any risk of ASD‐related diseases. A participant was used as a control if he/she had an AQ below 32, did not have any personal or family history of neurological disorders, psychiatric illness, or adverse pregnancy outcomes such as fetal loss, and had completed education through at least middle school to exclude any risk of low intellectual functioning.

### 2.3 Panel design

We selected syndromic genes as follows and grouped them by the strength of existing evidence, from strongest to weakest: Group 1, high‐confidence syndromic genes labeled as Levels 3 or 4 in AutismKB that were generally acknowledged to be related to ASD or reported in more than one family with ASD (Xu et al., 2012); Group 2, genes summarized by Neale et al. (2012) that were only reported in a single family or a single case with ASD; and Group 3, genes reported by recent studies of new syndromes possibly related to ASD (Hoppman‐Chaney, Wain, Seger, Superneau, & Hodge, 2013; Schaaf et al., 2013; Sweatt, 2013; Williams et al., 2010).

We selected nonsyndromic genes by ranking all candidate genes in AutismKB (Xu et al., 2012) using an improved multidimensional evidence‐based candidate gene prioritization approach. Specifically, we used a new benchmark gene set that included genes that had been reported more than three times in high‐quality literature studies, and we used the “Nelder–Mead” algorithm (Nelder & Mead, 1965) to optimize the weight matrix to ensure that 95% of the benchmark genes were ranked in the top 2% of all candidate genes. The genes with weighted combined scores greater than or equal to those of benchmark genes were classified as the core gene set. We then selected and grouped the nonsyndromic genes as follows: Level 1, genes belonging to the core gene set and supported by more than one genetic study; Level 2, the top 300 genes ranked by the above weighted combined scores or genes collected from AutDB (Basu, Kollu, & Banerjee‐Basu, 2009) and supported by more than one genetic study; Level 3, genes recently predicted by the TADA model (He et al., 2013) and genes reported by two genetic studies focused on the effect of homozygous variants on ASD (Lim et al., 2013; Yu et al., 2013). These three groups were further classified into “Level 1—Association only”, “Level 1—Association and other”, “Level 2—Association only”, “Level 2—Association and other”, and “Level 3—Association and other” according to whether the gene was supported only by association studies or by both association studies and other studies. In addition, 26 syndromic genes were also recurrently reported in nonsyndromic ASD patients and were selected.

To control for population structure, we added to the panel the top 300 highly differentiated ancestry informative markers (AIMs) from the published panel of AIMs for Han Chinese (Qin et al., 2014).

Probes were designed using the SSAHA algorithm to capture all exons of the selected genes, 50 bp of the intronic sequences flanking the exons, 2 kb of upstream sequences, and the 300 AIMs (Roche NimbleGen, Inc., Madison, WI).

### 2.4 Library preparation, targeted capture, and sequencing

Genomic DNA was extracted from blood samples of participants and sheared on a Covaris S220 (Covaris, Inc., Woburn, MA) to generate 200‐bp fragments. Sheared fragments were end‐repaired, A‐tailed, and adapter‐ligated using the KAPA LTP Library Preparation Kit (Kapa Biosystems, Wilmington, MA) according to the manufacturer's instructions. Dual‐SPRI size selection by Agencourt^®^ AMPure^®^ XP beads (Beckman Coulter, Inc., Brea, CA) was used to select approximately 340‐bp adapter‐ligated fragments. Pools of four amplified libraries were captured to the custom SeqCap^®^ EZ Library (Roche NimbleGen, Inc., Madison, WI) following the manufacturers’ instructions. Libraries were paired‐end sequenced (2 × 100 bp) using the Illumina HiSeq 2500 platform (Illumina Inc., San Diego, CA).

### 2.5 Variant calling

Adapter sequences were removed from raw reads using cutadapt. Reads for which >50% of bases had a base quality <6 and reads for which >10% of bases were “N” were removed. Clean reads were mapped to the human genome (GRCh37) using BWA (H. Li & Durbin, 2017) MEM (v0.7.10), processed with Picard (v1.117; http://broadinstitute.github.io/picard/) to mark duplicates, processed with GATK (McKenna et al., 2010) to realign around indels, recalibrate base quality scores, call variants, and filter variants according to the standard GATK pipeline (v3.4; Van der Auwera et al., 2016), and then annotated by ANNOVAR (K. Wang, Li, & Hakonarson, 2010). A variant was defined as an “LoF variant” if it was nonsense, essential splice site, or frameshift variant and as a “damaging missense variant” if it was predicted to be damaging by at least five out of nine prediction algorithms by dbNSFP v3.0b2a (X. Liu, Jian, & Boerwinkle, 2013; X. Liu, Jian, & Boerwinkle, 2001; SIFT predicted as “D”, PolyPhen2 HDIV score > 0.5, PolyPhen2 HVAR score > 0.5, LRT predicted as “D”, MutationTaster predicted as “A” or “D”, MutationAssessor predicted as “H” or “M”, FATHMM predicted as “D”, MetaSVM predicted as “D”, and MetaLR predicted as “D”). LoF and damaging missense variants were together labeled as “functional variants.”

To call copy number variants (CNVs), we ran XHMM 1.0 (Fromer & Purcell, 2014) using the following parameters to remove outlier samples and targets: minTargetSize = 100, maxTargetSize = 20,000, minMeanTargetRD = 10, maxMeanTargetRD = 600, minMeanSampleRD = 25, maxMeanSampleRD = 400, maxSdSampleRD = 150, maxSdTargetRD = 30, and PVE_mean_factor = 0.7. At the genotyping stage, the default parameters were used. CNVs were then filtered on five attributes: XHMM quality score (SQ) ≥ 60, estimated CNV length ≥ 1 kb, minor allele frequency (MAF) in our data < 1%, MAF in Database of Genomic Variants (DGV) Gold Standard variants (MacDonald, Ziman, Yuen, Feuk, & Scherer, 2010) < 1%, and encompassing the coding region of any gene.

### 2.6 Quality control

We excluded individuals whose recorded gender did not match that estimated by PLINK (Purcell et al., 2007); individuals with a cross‐sample contamination level >2%, as estimated by verifyBamID (Jun et al., 2012); and individuals with high levels of pairwise identity by descent (IBD)) with others, as estimated by PLINK (PI_HAT > 0.2); to remove contaminated samples and cryptic related samples. Additionally, we removed nucleotide positions that had >10% missing genotypes and positions that failed a Hardy–Weinberg equilibrium test (*p* < 0.001).

### 2.7 Validation of SNVs, indels, and CNVs

Single nucleotide variants (SNVs) and indels were validated via polymerase chain reaction (PCR)‐Sanger sequencing. Rare exonic autosomal CNVs were validated by SYBR‐based quantitative PCR (qPCR). Reference genes chosen from *COBL*, *GUSB*, *PPIA*, and *SNCA* were included based on the minimal coefficient of variation, and the normal control was assigned a value of 1 to normalize the data. The $2^{-\Delta \Delta C_{t}}$ relative quantification method (Livak & Schmittgen, 2001) was used to analyze the results. A CNV was validated if the normalized signal ratio showed an increase or decrease >25% in dosage compared with the calibrator sample, a pooled sample of male DNA (cat #G1471) (Promega, Madison, WI).

For the most frequent types of CNVs in cases, 15q13.3 duplications, we used the Infinium Global Screening Array‐24 v2.0 + Multi Disease microarray platform (GSAMD; Illumina Inc., San Diego, CA), which contains 759,993 probes, to further validate the CNVs and determine accurate boundaries. CNVs were called using the GenomeStudio v2011.1 (Illumina Inc., San Diego, CA) and cnvPartition v3.0.7 (Illumina Inc., San Diego, CA) software and filtered out if the confidence value was < 35 or the region was comprised of < 3 probes. A higher‐density chip, CytoScan High‐Density (HD; Affymetrix, Santa Clara, CA), which contains 2,696,550 probes, was used to further confirm the boundaries for the 15q13.3 duplication in AU032303. CNVs were called by Chromosome Analysis Suite (ChAS) software, and only regions ≥ 25 kb in size and comprising at least 25 contiguous probes were considered.

### 2.8 Classification criteria for pathogenic and likely pathogenic variants

Potential diseases‐causing variants were evaluated from three types of rare variants: syndromic variants, nonsyndromic variants, and CNVs. When analyzing the pathogenicity of rare variants located in syndromic genes, we used two criteria, our in‐house criteria and standards, to discover the pathogenic and likely pathogenic variants, as well as guidelines for the interpretation of sequence variants established by the American College of Medical Genetics and Genomics (ACMG) and the Association for Molecular Pathology (AMP) (ACMG–AMP guidelines) in 2015 (Richards et al., 2015), to confirm the classification.

First, a rare SNV or indel (MAF < 1%) in a syndromic gene was initially classified as pathogenic if: (a) the same variant was reported as pathogenic in both the Human Gene Mutation Database (HGMD; release 2016.3; Stenson et al., 2017) and ClinVar (Landrum et al., 2014); (b) there were DN LoF variants in the proband with no family history and the variant was located in a gene where LoF is a known mechanism of the syndrome; and likely pathogenic if: (a) the same variant was reported as likely pathogenic in HGMD or ClinVar; (b) the same amino acid change was reported as pathogenic, despite a different nucleotide change; (c) the inherited LoF variant is in a gene where LoF is a known mechanism of the syndrome. Furthermore, only when the above variants conformed to the inheritance pattern of the syndrome was the variant considered to be pathogenic or likely pathogenic. Second, ACMG–AMP guidelines were applied to further confirm the pathogenic and likely pathogenic variants as classified by the above in‐house criteria. First, we used the semiautomated tool InterVar (Q. Li & Wang, 2017). Second, we performed a manual check of InterVar's automated interpretation on 18 criteria and reviewed the evidence for the remaining 10 criteria. The benign and likely benign variants classified by ACMG–AMP were excluded when calculating the diagnostic yield of syndromic genes.

Considering the complexity of the genetic basis of nonsyndromic cases, we only considered DN LoF variants as likely pathogenic. If any DN LoF variant had been identified in other unrelated cases, the variant was considered pathogenic.

A rare CNV was considered likely pathogenic if it had been reported as pathogenic in ClinVar for autistic features or developmental delay. If the CNV had also been identified in more than one ASD patient, as recorded in AutismKB 2.0 (Yang et al., 2018), or identified in only one patient but as a DN variant, the CNV was considered pathogenic. The CNVs in ClinVar and AutismKB 2.0 were required to share at least one base with our CNVs of interest.

Parentage was confirmed in all cases with DN variants. The data regarding the above pathogenic and likely pathogenic variants were submitted to ClinVar (submission ID: SUB4367077, https://www.ncbi.nlm.nih.gov/clinvar/?term=SUB4367077). The nomenclature of the variants was based on the reference sequence: *IQSEC2* (NM_001111125.2), *MEF2C* (NM_002397.4), *MBD5* (NM_018328.4), *PTEN* (NM_001304717.2), *CDKL5* (NM_003159.2), *HEPACAM* (NM_152722.4), *NF1* (NM_001128147.2), *RNF135* (NM_032322.3), *SHANK3* (NM_033517.1), *TSC2* (NM_000548.4), *MAP2K1* (NM_002755.3), *SHANK2* (NM_133266.4), *NR3C2* (NM_000901.4), *SBF1* (NM_002972.3), *DEAF1* (NM_021008.3), and *CNTNAP2* (NM_014141.5). Nomenclature was checked using xMutalyzer 2.0.29 (Wildeman, van Ophuizen, den Dunnen, & Taschner, 2008).

### 2.9 Statistical analysis

After performing the quality control described above, 521 cases and 483 controls remained for subsequent analyses. As population structure has been reported to have an impact on the result of rare variant analyses of case–control studies (L. Liu et al., 2013), we conducted a principal components analysis to detect ancestry differences between cases and controls using EIGENSTRAT (Price et al., 2006) based on the genotype data for AIMs. Only the first eigenvector was statistically significant (*p* = 2.31 × 10^−8^), and the first two eigenvectors are plotted in Figure S1. To better minimize spurious associations, we still adjusted for population structure along the first 10 eigenvectors in the analyses. First, we investigated differences in the variant spectrum stratified by gene groups and MAF between cases and controls. We built the logistic regression models using the phenotype (case: 1 and control: 0) as the dependent variable, and whether the patient carried the variant (he/she carried the variant: 1 and he/she did not carry the variant: 0), gender, and the first 10 eigenvectors from EIGENSTRAT as independent variables to test the difference in the fraction of samples carrying specific variant sets.

Furthermore, we studied the clinical features of the cases with *SHANK3* and *SHANK2* variants and 15q11–13 duplications to explore their common characteristics and then conducted genotype–phenotype correlation analyses, focusing on loss of language skills, minimal verbal skills, unusual sensory interests, self‐injurious behavior, epilepsy or tics, gastrointestinal problems, hypotonia, and insensitivity to pain. By comparing the variant spectra between cases with and without specific features using Fisher's exact test with a 2 × *m* contingency table, where “*m*” indicates the number of genes, we selected the feature shown to be marginally statistically significant to investigate the correlation with the gene by Fisher's exact test with a 2 × 2 contingency table.

All statistical analyses were conducted using R 3.2.2 (R Core Team, 2015). For all analyses, *p* < 0.05 were considered statistically significant. However, when multiple tests were performed, the Bonferroni correction was used to adjust *p* values by multiplying each *p* value by the total number of tests. In this study, the following three questions utilized multiple testing: The difference between cases and controls in the fraction of cases carrying variants in five nonsyndromic and three syndromic gene subgroups and the difference in variant spectra between cases with and without specific features. Therefore, only these with adjusted *p* < 0.05 were considered statistically significant.

## 3 RESULTS

### 3.1 The most comprehensive ASD‐targeted resequencing panel to date

**Figure 1:**
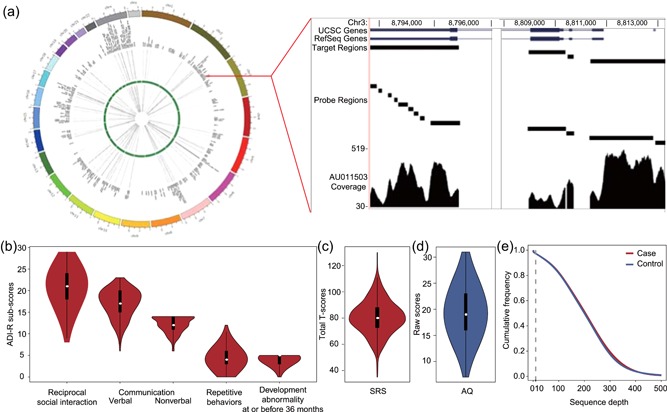
Characteristics of the targeted panel and the case–control cohort. (a) The sequencing depths of targeted genes for 1,004 qualified samples are summarized in heatmaps inside the Circosmap, with cases and controls shown outside and inside the green circle, respectively. The targeted resequencing depths are proportional to the color depth. The right panel illustrates the capture sequencing details of one region of interest in case AU011503 as an example. (b) The raw score distributions of ADI‐R content areas (quality of social interaction; communication and language; repetitive, restricted, and stereotyped interests and behavior; and evidence of onset of the disorder by 36 months of age) for the cases are represented by violin plots. The y‐axis shows the raw score of each ADI‐R subdomain. The ADI‐R‐specified cutoffs for these subdomains were 10, 8, 7, 3, and 1, respectively. (c) The distribution of the SRS total *T*‐scores of cases is shown as a violin plot. (d) The distribution of the AQ raw scores of controls, for whom the scores were below the cutoff of 32. (e) Cumulative frequency distribution of the sequencing depth in all 1,004 samples. The read depth is consistent across both cases and controls. ADI‐R: Autism Diagnostic Interview‐Revised; AQ: Adult Autism Spectrum Quotient; SRS: Social Responsiveness Scale

Our gene panel contained 358 genes and 300 AIMs. A total of 10,470 regions were targeted for a final capture size of 4.74 Mb (Figure 1a). Specifically, the panel contained 111 syndromic genes, including 78 Group 1 genes, 29 Group 2 genes, and 4 Group 3 genes (Table S2), as well as 247 nonsyndromic genes, including 58 “Level 1—Association only” genes, 54 “Level 1—Association and other” genes, 26 “Level 2—Association only” genes, 36 “Level 2—Association and other” genes, and 73 “Level 3—Association and other” genes (Table S3).

### 3.2 The largest Chinese ASD cohort assessed by ADI‐R and ADOS to date

A total of 539 Han Chinese children affected by ASD (male: 87.38%, age: 4.92 ± 1.20 years old) and 512 Han Chinese controls (male: 76.17%, age: 29.77 ± 9.12 years old) were studied. All cases were diagnosed as ASD by the ADI‐R (Lord et al., 1994; Figure 1b), most with the ADOS (Lord et al., 1989) and Social Responsiveness Scale (SRS; Constantino & Gruber, 2014) to evaluate their extent of autistic social impairment (Figure 1c). All controls were screened for ASD by measurement of their AQ (Baron‐Cohen et al., 2001). The AQ scores for all controls were below 32, and their distribution is shown in Figure 1d. Furthermore, according to their self‐report, the controls did not have any personal or family history of neurological disorders or psychiatric illness related to ASD, such as schizophrenia, attention deficit hyperactivity disorder, and mental retardation; did not have adverse pregnancy history, such as stillbirth; and completed middle school education, to ensure normal intelligence.

### 3.3 High‐quality target capture and resequencing of 358 genes

For each case and control, the target regions were captured by our ASD panel and resequenced. After quality control, 521 cases and 483 controls remained for downstream analyses. On average, 13.13 million clean reads were generated per individual and 4.93% of reads were duplicates and were removed. Of the remaining reads, 99.99–100% were aligned to the human genome, and an average of 72.50% of reads were aligned to the target regions. This highly efficient sequencing panel resulted in an average depth of 204.25X in targeted regions, and 97.31% of the targeted bases were covered with depth >10X. Cases and controls had similar technical sequencing metrics (Table S4), including coverage depth (*p* = 0.64, Wilcoxon's rank‐sum test; Figure 1e).

### 3.4 Statistically significant differences in ultra‐rare functional variants between cases and controls

**Figure 2:**
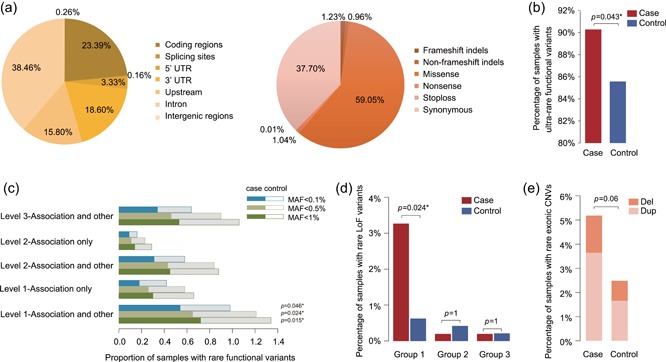
Variant spectra and differences between cases and controls. (a) The distribution of different types of variants for all targeted regions (left pie chart) and the targeted coding regions (right pie chart). (b) The percentage of cases (red bar) with ultra‐rare functional variants in 358 genes was significantly higher than that in controls (blue bar) (*p* = 0.043 by logistic regression). (c) The differences in the proportion of rare variants between cases and controls for five subgroups of nonsyndromic genes. The bars represent the proportions of cases (solid bars) and controls (hollow bars) that carried the corresponding variants, as grouped by subgroups and MAF. Only rare functional variants in the “Level 1—Association and other” gene set were significantly enriched in cases when compared with the controls. The adjusted *p* values after Bonferroni correction are shown on the right of the bars. (d) Comparison of the percentage of samples with rare LoF variants in syndromic genes following their inheritance patterns in cases (red bar) and controls (blue bar) grouped by subgroups. Only the variants in the Group 1 gene set were detected in more cases than controls, and the difference was statistically significant (adjusted *p* = 0.024 by logistic regression after Bonferroni correction). (e) Comparison of rare autosomal exonic CNVs detected in cases and controls. The proportion of samples with deletions is shown in the upper bar and that of duplications is shown in the lower bar. CNV: copy number variant; MAF: minor allele frequency

We identified a total of 45,847 variants, whose distribution is shown in Figure 2a. Ultra‐rare (i.e., MAF < 0.1%) functional variants were found in significantly more cases than controls, as shown by logistic regression after controlling for gender and population structure by including the first 10 eigenvectors (*p* = 0.043; Figure 2b), whereas the total number of all variants, the total number of functional variants, and the number of functional variants with a MAF ≥ 0.1% were similar between the cases and controls (*p* = 1, 1, and 1, respectively). Details about all SNVs and indels are provided in Table S5.

For nonsyndromic genes, functional variants with a MAF < 0.1% were significantly enriched in cases when compared with controls, as estimated by the proportion of carriers among cases and controls using logistic regression, correcting for gender and population structure (*p* = 0.039). We then tested the enrichment in five subgroups. After Bonferroni correction, only rare functional variants in the “Level 1—Association and other” group were significantly enriched in cases compared with controls (Figure 2c). On the contrary, there was no significant difference in the spectrum of “Level 1—Association only” variants between cases and controls, although this gene set also belonged to the “Level 1” class. Additionally, controls carried even more rare functional variants in the genes of the “Level 1—Association only” class than cases. The result of this comparison with the “Level 2—Association only” gene set was similar to that of the “Level 1—Association only” set, and the number of individuals with rare functional variants in both of these two gene sets was less than those in the other three subgroups. Considering that the designation of these two gene sets was only supported by genome‐wide or candidate gene association studies, these genes may influence the risk of ASD mainly through common variants, not rare variants. The variant spectra of the “Level 2—Association and other” and “Level 3—Association and other” subgroups showed very weak differentiation between cases and controls, suggesting that the relationship between these genes and ASD requires further confirmation. Thus, among all of the subgroups, “Level 1—Association only” displayed the highest diagnostic potential and should be given first priority in genetic testing for ASD patients.

For syndromic genes, cases harbored significantly more rare LoF variants (MAF < 1%) following the inheritance pattern of the associated syndrome than the controls (19 cases vs. 6 controls, logistic regression correcting for gender and population structure, *p* = 0.023). All of these variants were validated by Sanger sequencing. This significant difference was mainly due to “Group 1” genes, indicating that this subgroup of genes was the most discriminative (Figure 2d). The other subgroups, “Group 2” and “Group 3,” displayed limited abilities to differentiate cases and controls. This suggested that “Group 1” genes have higher diagnostic potential and could be used to simultaneously screen for multiple ASD‐related syndromes in a clinical setting.

In addition to SNVs, 42 rare autosomal exonic CNVs were identified in 27 cases and 12 controls and were validated by qPCR. There were two cases that carried two CNVs. A larger percentage of cases harbored CNVs than controls, as estimated by the logistic regression correcting for gender and population structure (*p* = 0.060), but did not reach the significance threshold (Figure 2e). Eight cases and two controls carried CNVs encompassing syndromic genes. Previous studies have shown that the most frequently and consistently reported chromosomal abnormalities in ASD patients, as detected by karyotyping, fluorescence in situ hybridization (FISH), and CMA, are 15q11–13, 16p11.2, and 22q11.2 CNVs (Abrahams et al., 2013; Schaefer & Guidelines, 2008, 2013; Yang et al., 2018). In our cohort, five cases and only one control carried CNVs located in these regions. Among them, 15q11–13 duplications were detected most frequently (four cases).

### 3.5 Pathogenic or likely pathogenic variants with potential diagnostic value were found in 9.5% of cases

**Figure 3:**
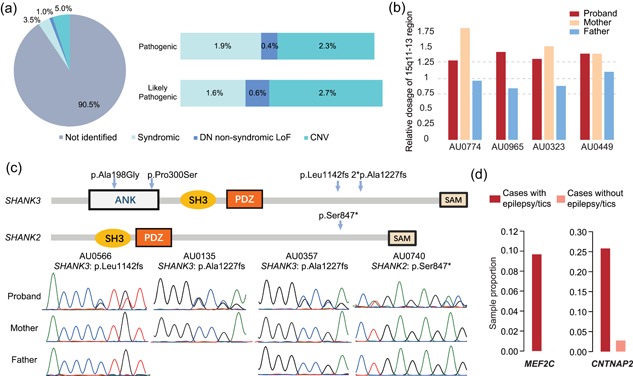
Pathogenic and likely pathogenic variants and phenotype–genotype correlations. (a) Proportion of cases with pathogenic and likely pathogenic SNVs and indels in syndromic and nonsyndromic genes as well as CNVs detected by our panel are summarized in the left pie chart. The proportions of cases carrying pathogenic and likely pathogenic variants of each type are shown in the bars on the right. Pathogenicity determination results for syndromic variants were based on our criteria after excluding variants of *CDKL5*, which had been classified as likely benign by the ACMG–AMP guidelines. (b) Four 15q11–13 duplication events that were validated by qPCR. Three variants were inherited from the mothers. The DNA sample of AU096503′s mother was unavailable. (c) Locations of five variants in *SHANK3* and *SHANK2* with protein domains as annotated by InterPro are shown in the upper panel, and Sanger sequencing chromatograms of four pathogenic variants are shown in the lower panel. Two unrelated cases carried the same *SHANK3* frameshift variant (p.Ala1227fs). (d) The proportions of cases with epilepsy/tics carrying rare functional variants in *MEF2C* and *CNTNAP2* were higher than that of cases without epilepsy/tics. ACMG: American College of Medical Genetics and Genomics; AMP: Association for Molecular Pathology; CNV: copy number variants; qPCR: quantitative polymerase chain reaction

To assess the diagnostic yield of our panel, we assessed the pathogenicity of identified rare variants via two methods: our criteria and stringent ACMG–AMP guidelines (Kearney et al., 2011; Richards et al., 2015). For syndromic genes, we first classified eight pathogenic variants and seven likely pathogenic variants in 11 genes. Of these, five variants (31.33%) had been previously reported as pathogenic, and the remaining variants (66.67%) were novel or were a different nucleotide change but encoded the same amino acid change as previously reported pathogenic variants. After being reanalyzed using ACMG–AMP guidelines, seven variants were classified into different categories compared with using our in‐house criteria. One variant, *CDKL5* c.2854C>T (p.Arg952*), was classified as likely benign. Three variants, *IQSEC2* c.1229delC (p.Pro410fs), *PTEN* c.404dupG (p.Arg135fs), and *PTEN* c.457dupC (p.Lys152fs), were upgraded from likely pathogenic to pathogenic. Three variants, *HEPACAM* c.803+1G>A, *NF1* c.1737dupT (p.Tyr579fs) and *RNF135* c.1014delG (p.Gln338fs), were downgraded from likely pathogenic/pathogenic to uncertain significance. After excluding one likely benign variant in *CDKL5* c.2854C>T (p.Arg952*), 14 rare variants spanning 10 genes were classified as pathogenic or likely pathogenic in 18 cases (Tables 1 and S6). These variants accounted for 3.5% of cases (Figure 3a). All but one of these variants belonged to the Group 1 gene set. The pathogenic variants of *SHANK3* and *MEF2C* were the most frequent, including five LoF variants, of which four were DN and one was of unknown origin.

**Table 1:** Summary of pathogenic and likely pathogenic variants in syndromic genes

Samples	Sex	cDNA	Protein	Genotype	Gene	Gene category	Type	Origin	Classification	ACMG classification
AU076603	F	c.1229delC	p.Pro410fs	het	*IQSEC2*	Group 1	fs del	Unknown	LP	P
AU065903	M	c.766C>T	p.Arg256*	het	*MEF2C*	Group 1	Nonsense	*De novo*	P	P
AU049703	M	c.403–1G>T	–	het	*MEF2C*	Group 1	Splicing	*De novo*	P	P
AU012204	M	c.973C>T	p.Arg325*	het	*MBD5*	Group 1	Nonsense	*De novo*	P	P
AU060803	M	c.404dupG	p.Gly136fs	het	*PTEN*	Group 1	fs ins	Paternal	LP	P
AU037503	M	c.460dupC	p.Arg154fs	het	*PTEN*	Group 1	fs ins	Maternal	LP	P
AU095803	M	c.2854C>T	p.Arg952*	hom	*CDKL5*	Group 1	Nonsense	Maternal	LP	Likely benign
AU048503	M	c.803+1G>A	–	het	*HEPACAM*	Group 2	Splicing	Paternal	LP	VUS
AU065403	M	c.1742dupT	p.Leu581fs	het	*NF1*	Group 1	fs ins	Maternal	LP	VUS
AU065503	M	c.1742dupT	p.Leu581fs	het	*NF1*	Group 1	fs ins	Maternal	LP	VUS
AU099703	M	c.1742dupT	p.Leu581fs	het	*NF1*	Group 1	fs ins	Maternal	LP	VUS
AU052603	M	c.1015delG	p.Val339fs	het	*RNF135*	Group 1	fs del	Paternal	P	VUS
AU095503	M	c.1015delG	p.Val339fs	het	*RNF135*	Group 1	fs del	Paternal	P	VUS
AU056603	F	c.3424_3425del	p.Leu1142fs	het	*SHANK3*	Group 1	fs del	*De novo*	P	P
AU013503	F	c.3679dupG	p.Ala1227fs	het	*SHANK3*	Group 1	fs ins	Unknown	P	P
AU035703	F	c.3679dupG	p.Ala1227fs	het	*SHANK3*	Group 1	fs ins	*De novo*	P	P
AU039303	M	c.4753_4763del	p.Lys1585fs	het	*TSC2*	Group 1	fs del	*De novo*	P	P
AU018703	M	c.199G>A	p.Asp67Asn	het	*MAP2K1*	Group 1	Missense	Unknown	P	P
AU017403	M	c.1081C>G	p.Leu361Val	het	*TSC2*	Group 1	Missense	Maternal	LP	LP

*Note*. RefSeq sequences used: *IQSEC2* (NM_001111125.2), *MEF2C* (NM_002397.4), *MBD5* (NM_018328.4), *PTEN* (NM_001304717.2), *CDKL5* (NM_003159.2), *HEPACAM* (NM_152722.4), *NF1* (NM_001128147.2), *RNF135* (NM_032322.3), *SHANK3* (NM_033517.1), TSC2 (NM_000548.4), *MAP2K1* (NM_002755.3).

F: female; M: male; cDNA: complementary DNA; fs del: frameshift deletion, fs ins: frameshift insertion, LP: likely pathogenic; P: pathogenic; VUS: variants of uncertain significance.

For the nonsyndromic genes, among the 123 ultra‐rare LoF variants (MAF < 0.1%) validated by Sanger sequencing, the parental origin of 83 variants could be confirmed. Of these, five were DN, including a frameshift deletion of *SHANK2* (c.2540_2541del [p.Ser847*]), nonsense variants of *NR3C2* (c.1609C>T [p.Arg537*]) and *SBF1* (c.1180G>T [p.Glu394*]), and splice variants of *DEAF1* (c.664+2T>G) and *CNTNAP2* (c.1778–1G>C). The frameshift deletion of *SHANK2* had also been detected in a proband from the SSC. For *NR3C2*, a DN LoF variant and two DN missense variants had previously been reported in probands from the SSC. Combining the findings from our samples and the SSC, *NR3C2* was upgraded to a candidate gene with high reliability. According to our criteria, the two DN LoF variants of *SHANK2* and *NR3C2* were considered pathogenic, and the other three variants were considered likely pathogenic. Thus, in total, DN LoF variants of nonsyndromic genes accounted for approximately 1% of cases in our samples (Figure 3a).

In addition to SNVs, 28 rare exonic CNVs were also classified as pathogenic or likely pathogenic, accounting for another 5% of cases (Table 2 and Figure 3a). Of these, seven CNVs (25%) were DN. Duplications of 15q11–13 occurred in four cases, especially those in 15q13.3, which were found in three cases. GSAMD and CytoScan HD further confirmed and refined the boundaries and size of the 15q13.3 duplications (Table S7). The 15q13.3 duplications were found to be 375–514 kb, only encompassing *CHRNA7* and the first exon of *OTUD7A* (Figure S2). Except for AU096503, whose maternal sample was not available, all carriers inherited the duplications from their mothers (Figure 3b). Large maternal duplication of 15q11–13 has been found to be the most frequent variant in ASD in previous studies, accounting for approximately 1–2% patients, as detected by karyotyping, FISH, and CMA (Huguet, Ey, & Bourgeron, 2013; Sutcliffe, Nurmi, & Lombroso, 2003). Duplication and deletion of 15q13.3 are also CNV hotspots in ASD. The *CHRNA7* gene, representing the smallest overlapping region of all of the 15q13.3 deletions and duplications, has been suggested as a candidate gene responsible for the cognitive and behavioral abnormality of 15q13.3 CNVs (Gillentine & Schaaf, 2015). Although 15q13.3 duplications have decreased penetrance when compared with deletions, there is evidence suggesting that they may be pathogenic (Szafranski et al., 2010). Furthermore, ASD occurs more frequently in individuals with *CHRNA7* duplications than in those with deletions (Gillentine & Schaaf, 2015). For this dosage‐sensitive region, our panel only included the *CHRNA7* gene. With this panel alone, we detected 15q13.3 microduplications in 0.58% of cases and no controls. Thus, considering that small changes were more likely to be missed by CMA because their probe density are relatively sparse, small CNVs in this region may be more frequent than previously estimated, especially in Chinese cohorts. Thus, 15q13.3 microduplications merit more consideration in genetic testing for ASD.

**Table 2:** Summary of identified pathogenic and likely pathogenic copy number variants

Samples	Sex	Band	Predicted position	Type	Size (kb)	Targeted genes	Origin	Classification
AU052603	M	1q41	1:216219723‐216251754	dup	32	*USH2A*	*De novo*	LP
AU021603	M	1q42.2	1:231885622‐231955040	del	69	*DISC1*	Maternal	P
AU074903	M	2q14.3	2:128324168‐128335911	dup	12	*MYO7B*	Maternal	LP
AU096303	M	2q24.3	2:166164308‐166211231	del	47	*SCN2A*	Inherited	LP
AU042703	F	2q37.3	2:239969814‐240325346	del	356	*HDAC4*	Paternal	P
AU061503	M	4q35.2	4:187560826‐187649850	del	89	*FAT1*	Paternal	LP
AU053003	M	5p15.31	5:9318434‐9380185	dup	62	*SEMA5A*	*De novo*	LP
AU052603	M	6p22.3	6:15452187‐15487823	dup	36	*JARID2*	*De novo*	LP
AU052703	M	6p22.3	6:15452187‐15487823	dup	36	*JARID2*	*De novo*	LP
AU066703	M	6q23.3	6:135778582‐135784494	del	6	*AHI1*	Maternal	LP
AU050103	M	7q31.1	7:107788021‐107834922	dup	47	*NRCAM*	Paternal	LP
AU083003	M	7q31.32	7:122753538‐122769585	del	16	*SLC13A1*	Maternal	P
AU048603	F	7q32.3	7:131829828‐131908450	dup	79	*PLXNA4*	Unknown	LP
AU049703	M	7q32.3	7:131844182‐131883445	dup	39	*PLXNA4*	*De novo*	P
AU037803	M	7q35	7:145811453‐146537046	dup	726	*CNTNAP2*	*De novo*	P
AU027103	M	8q24.13	8:124810280‐124827740	dup	17	*FAM91A1*	Maternal	LP
AU036305	M	8q24.13	8:124810280‐124827740	dup	17	*FAM91A1*	Maternal	LP
AU090603	M	8q24.3	8:145057050‐145058649	dup	2	*PARP10*	Maternal	LP
AU051103	M	9p24.1	9:6755641‐7128286	del	373	*KDM4C*	Maternal	P
AU049803	M	10q21.3	10:69407123‐69457949	dup	51	*CTNNA3*	Paternal	LP
AU077403	M	15q11.2–12	15:25074895‐27186686	dup	2112	*ATP10A, GABRB3, SNRPN, UBE3A*	Maternal	P
AU096503	M	15q13.3	15:32320686‐32455586	dup	135	*CHRNA7*	Unknown	P
AU032303	M	15q13.3	15:32320686‐32462434	dup	142	*CHRNA7*	Maternal	P
AU044903	M	15q13.3	15:32320686‐32462434	dup	142	*CHRNA7*	Maternal	P
AU078903	M	15q25.3	15:88669452‐88801962	dup	133	*NTRK3*	Maternal	P
AU053003	M	16q24.1	16:85667470‐85709862	dup	42	*GSE1*	Inherited	LP
AU035503	M	20p12.1	20:13974146‐14034142	dup	60	*MACROD2*	Unknown	LP
AU033603	F	22q11.21	22:19742226‐19957548	del	215	*COMT, GNB1L, TBX1*	*De novo*	P

*Note*. LP: likely pathogenic; P: pathogenic.

In addition to defining strict classification criteria, we investigated the carriers’ clinical phenotypes to further confirm the pathogenicity of the above variants. In general, carriers showed highly consistent genotype–phenotype correlations, particularly for the pathogenic variants (Table S8). For example, in *MEF2C*, we detected two pathogenic variants in two unrelated cases. Heterozygous variants of *MEF2C* can result in autosomal dominant mental retardation 20 (MRD20), which is mainly characterized by severe mental retardation, absence of speech, epilepsy, and autistic behavior (Nowakowska et al., 2010). Some MRD20 cases also display hypotonia, delayed motor development, variable dysmorphic features, and variable brain anomalies on imaging. These two cases displayed the typical symptoms described above. Additionally, we identified a pathogenic variant of *TSC2* in AU039303, who was rediagnosed with tuberous sclerosis and epilepsy. For commonly reported CNVs, such as 15q11–13 duplication, 2q37 deletion, and 22q11.2 deletion, we also found that many features of our carriers were consistent with previously reported phenotypes of the cases carrying these CNVs (Table S8). Thus, considering the severity of the variants and the clinical phenotypes of carriers, our panel detected pathogenic and likely pathogenic variants in approximately 9.5% of ASD cases (Figure 3a).

### 3.6 Genotype–phenotype correlations in ASD cases

In this study, pathogenic variants of *SHANK3* and *SHANK2* showed the highest incidence. In addition to the four above LoF variants, another two missense variants of *SHANK3*, c.593C>G (p.Ala198Gly) and c.898C>T (p.Pro300Ser), which have been reported in Caucasian ASD patients, were found in two cases (Figure 3c). In total, these six variants explained 1.2% of cases. Although the association between ASD and *SHANK* genes has been established, the phenotype of the carriers requires further exploration. We conducted a clinical investigation of the six carriers described above (Table S9). All showed severe language and social deficits, which were consistent with previous findings in Caucasian patients (Moessner et al., 2007). Additional characteristics enriched in this group included sleep disorders (83.3%) and abnormal gait (66.7%). All LoF variants of *SHANK3* were found in female cases. Our results suggest preferential screening for pathogenic variants in *SHANK3* in female patients with the above‐mentioned characteristics.

Small duplications in 15q11–13 were another frequent type of variant in our cases, occurring recurrently in 0.8% of cases. For these four carriers, we also extracted 24 clinical characteristics and conducted a phenotype analysis (Table S10). Previous studies found that individuals carrying 15q11–13 large duplications usually presented with mental retardation, epilepsy, and language impairment or loss, and many had hypotonia (Al Ageeli et al., 2014; Conant et al., 2014). These four carriers were all male and showed mental retardation, delayed language development, and severe autistic behavior. Three of the carriers had unusual sensory interests and a family history of autism‐related diseases. These carriers included AU096503, who had a family history of mental retardation; AU032303, who had a family history of developmental delay; and AU044903, who had a family history of delayed language developmental and epilepsy.

In addition to our comprehensive analysis of the most frequently occurring genes and CNV regions in our samples, we also aimed to identify common genetic factors underlying eight comorbid conditions, including loss of language skills, minimal verbal skills, unusual sensory interests, self‐injurious behavior, epilepsy or tics, gastrointestinal problems, hypotonia, and insensitivity to pain, via a two‐step method. First, we explored whether cases having specific comorbidity displayed a difference in their variant spectrum by Fisher's exact test with a 2 × *m* contingency table counting the number of variants, where “*m*” was the number of genes. As a result, of eight comorbidities, only “epilepsy/tics” showed marginal significance (Table S11). Next, we further explored which genes were correlated with “epilepsy/tics” using Fisher's exact test with a 2 × 2 contingency table. After Bonferroni correction, variants in two genes, *CNTNAP2* and *MEF2C*, were found to be significantly correlated with this phenotype (Figure 3d). In ASD cases with epilepsy or tics, 25.81% and 9.68% carried rare functional variants in *CNTNAP2* and *MEF2C*, respectively, whereas in ASD cases without epilepsy or tics, only 2.78% and 0% carried rare functional variants in *CNTNAP2* and *MEF2C*, respectively. Both *CNTNAP2* and *MEF2C* are syndromic ASD genes, causing Pitt–Hopkins‐like syndrome 1 (PTHSL1; Zweier et al., 2009) and chromosome 5q14.3 deletion syndrome (Le Meur et al., 2009), respectively. In addition to typical symptoms, most patients with these two disorders have epilepsy (Le Meur et al., 2009; Strauss et al., 2006). However, none of the *CNTNAP2* variants carried by the eight patients are sufficient to cause PTHSL1 because they are heterozygous and do not follow autosomal recessive inheritance. Similarly, except for those found in AU065903 and AU049703, the *MEF2C* variants carried in our samples also cannot be classified as pathogenic or likely pathogenic according to our criteria. Even so, our statistical analysis indicated that these variants still contributed to the epilepsy/tics comorbidity. We are the first to show that rare functional variants of *CNTNAP2* and *MEF2C* could significantly increase the risk of comorbid epilepsy/tics in nonsyndromic ASD patients, which should have a significant impact on ASD patient management.

## 4 DISCUSSION

In this study, we investigated the variant spectra, diagnostic yields, and genotype–phenotype correlations of 358 ASD candidate genes in a Chinese case–control cohort. Moreover, we updated the contribution estimate of these targeted genes to ASD. “Group 1” syndromic genes and “Level 1—Association and other” nonsyndromic genes were found to be most significantly associated with ASD and should be given high priority when screening ASD patients. Pathogenic and likely pathogenic variants were identified in 9.5% of cases. Variants in members of the *SHANK* gene family and 15q11–13 duplications were the most frequent abnormalities found in our Chinese ASD patients. New phenotype–genotype correlations were also identified. ASD patients carrying rare functional variants of *CNTNAP2* or *MEF2C* were more likely to have epilepsy/tics and require monitoring of these comorbidities. Our findings may facilitate genetic testing for ASD, especially in Chinese patients.

To our knowledge, our ASD‐targeted resequencing panel is the most comprehensive to date. To discover new ASD candidate genes, other targeted resequencing research on ASD cases (Alvarez‐Mora et al., 2016; Griswold et al., 2015; O’Roak et al., 2012, 2014; Stessman et al., 2017) selected target genes from GWAS‐based results (Griswold et al., 2015), from candidates identified by exome sequencing studies focused on DN variants (O’Roak et al., 2012), or from neurodevelopmental disorder risk genes (O’Roak et al., 2014; Stessman et al., 2017). These panels were comprised of <100 genes (Alvarez‐Mora et al., 2016; O’Roak et al., 2012, 2014). We considered a variety of genetic evidence regarding ASD and used machine‐learning methods to rank the genes and to further divide them into different groups by strength. Our panel covered 4.74 Mb of the genome and included not only 111 syndromic genes, but also 247 nonsyndromic genes. When considering the capture methods, we chose the Roche NimbleGen SeqCap EZ platform for panel development due to its high‐density tiling design of capture probes across targeted regions. Due to this high probe redundancy, our panel showed better enrichment and uniformity. In addition, we obtained data with average depth of 204.25X in targeted regions, which was much greater than that of other studies, all of who reported average depth <100X (Alvarez‐Mora et al., 2016; Griswold et al., 2015; O’Roak et al., 2012; Stessman et al., 2017). Higher sequencing depth also improved the reliability of variant identification.

Our sample sets included the only Chinese ASD cohort assessed with ADI‐R and ADOS by evaluators with certified reliability; these assessments are regarded as the “gold standard” of ASD diagnosis. Rediagnosis based on these uniform criteria allows us to effectively eliminate bias in the patients’ diagnoses obtained from different hospitals and at different times. Moreover, our controls were also screened for ASD by measuring AQ, and we excluded samples with any individual or family history of neurological disorders or psychiatric illness related to ASD and any risk of low intellectual function. Although cases and controls were not age matched, germline variants are generally considered not to change with age, unlike somatic mutations, DNA methylation, and RNA expression. In addition, for ASD, an early onset disorder, age is not an important factor influencing case or control enrollment. Thus, the age difference of the cases and controls should have a very limited impact on the study results.

Because our resequencing panel was designed using evidence from published genetic studies, it did not enable the expansion of current knowledge regarding causes of ASD. However, its comprehensive design allowed us to evaluate the differences in various gene sets between cases and controls. We observed a greater enrichment of rare variants of “Group 1” syndromic genes and “Level 1—Association and other” nonsyndromic genes in cases than in other subgroups. This further confirmed their strong associations with ASD in Chinese patients and suggested that they have a higher diagnostic potential.

With the development of research on ASD genetics and the increased attention paid to this disorder, there has been an increase in the number of patients referred for clinical genetic evaluation to identify the genetic etiology. A tiered genetic evaluation is recommended for ASD patients and CMA has been suggested as the first‐tier testing approach for CNVs (Schaefer et al., 2013; Shen et al., 2010). However, there is currently still no high‐throughput second‐tier testing to efficiently and economically screen for disease‐causing SNVs, although genomic technologies have advanced rapidly, and multiple genetic factors associated with ASD have been reported. Here, we used targeted resequencing to enrich for the highest‐confidence ASD candidate genes with clinical potential. Compared with whole‐genome and whole‐exome sequencing, targeted resequencing can more effectively discover variants of genes of interest at a lower cost and higher sequencing depth, although at the expense of the ability to identify variants outside of the target regions. We assessed the possible pathogenicity of the variants found in our cases using our criteria in combination with ACMG–AMP variant interpretation guidelines. Finally, pathogenic or likely pathogenic variants of targeted genes were identified in 9.5% of cases.

As our panel and analysis pipeline reliably detected rare SNVs, indels, and exonic CNVs of various gene sets simultaneously, we were able to further evaluate the contribution of different types of genetic variation to ASD. Pathogenic and likely pathogenic SNVs of syndromic ASD genes were identified in 3.5% of cases. However, this detection rate is lower than the expected yield. None of the syndromic ASD genes could explain more than 1–2% of the ASD cases, but collectively, they are estimated to be found in approximately 5% of the total ASD population (de la Torre‐Ubieta et al., 2005; Sztainberg & Zoghbi, 2016). This may be due to the fact that our patient population, recruited from training centers, tends to be enriched with nonsyndromic patients, as patients with syndromes always have more severe clinical features and may be rejected by the general ASD training centers. Another key potential reason for this difference is that our method cannot detect expansion of CGG repeats in the causal gene of fragile X syndrome, *FMR1*. Regardless, our method can still facilitate the evaluation of the proband and family for expected clinical features, which is especially important for the differential diagnosis of ASD cases. In contrast, pathogenic and likely pathogenic variants of nonsyndromic genes accounted for approximately 1% of our cases. In contrast to syndromes‐related ASD, the causality of nonsyndromic ASD is more complex and mostly attributed to multiple factors (Caglayan, 2010; de la Torre‐Ubieta et al., 2005). Thus, when performing a pathogenicity assessment, we only considered the DN LoF variants because they are generally considered to be the most damaging. As a portion of the variants’ origins was uncertain because the parental samples were unavailable, this diagnostic yield is likely underestimated. In addition to SNVs, pathogenic and likely pathogenic CNVs accounted for another approximately 5% of cases. Conventional technologies for CNV detection are more likely to overlook smaller CNVs. Here, we found that our panel can detect these smaller CNVs in targeted genes and can be used as a complement to genome‐wide large CNV detection. Considering that our cases may be relatively less likely to obtain abnormal chromosomal results because they generally did not have any other complicating abnormalities, such as dysmorphic features, which are known to have a high rate of detection for CNVs, we could expect that more CNVs in targeted genes would be identified in clinical patients.

For identifying the actual causal variants from those detected in the panel, we performed extensive analyses for variant interpretation and classification. We used our in‐house criteria to identify putative pathogenic and likely pathogenic variants of syndromic and nonsyndromic genes and CNVs. This criteria integrated variant determination protocols primarily based on those described in previously published studies or those collected from two databases, HGMD (Stenson et al., 2017) and ClinVar (Landrum et al., 2014). The ACMG–AMP guidelines (Richards et al., 2015) are more stringent when used to interpret the variants identified in genes that cause Mendelian disorders. As ASD‐related syndromes are all Mendelian disorders, we used this guideline to further confirm the pathogenic and likely pathogenic variants in syndromic genes. Seven variants were differentially classified by our criteria and the ACMG–AMP guidelines. We double‐checked the reasons for different classifications of these seven variants. The pathogenicity of three variants, *IQSEC2* c.1229delC (p.Pro410fs), *PTEN* c.404dupG (p.Gly136fs), and *PTEN* c.460dupC (p.Arg154fs), was upgraded. This was mainly because the population allele frequencies for these variants were extremely low, and the corresponding clinical features of the proband matched to the syndrome, which strengthened the evidence of pathogenicity. There were two variants, *HEPACAM* c.803+1G>A and *NF1* c.1742dupT (p.Leu581fs), that were downgraded from likely pathogenic to uncertain significance, and one variant, *RNF135* c.1015delG (p.Val339fs), was downgraded from pathogenic to uncertain significance. For the splice variant of *HEPACAM*, null variants in this gene are not a known mechanism of pathogenicity, which explains the loss of PVS (pathogenic very strong) evidence in the ACMG–AMP guidelines. Actually, *HEPACAM* was in the Group 2 gene set, which had less genetic evidence as the ASD candidate genes. For two other variants, we did not observe similar conditions in the parents who passed the variants on to their children, and they were considered healthy adults, which weakened the evidence of pathogenicity. The *CDKL5* c.2854C>T (p.Arg952*) variant was downgraded from likely pathogenic to likely benign. When applying our initial criteria, we collected the HGMD evidence with the tag of “DM?” and estimated it to be likely pathogenic. When applying the ACMG–AMP guidelines, we referred to the original study (Intusoma et al., 2011) and checked the computational prediction results, which were based on multiple tools, and added evidence for a designation of benign. The pathogenicity of this variant needs to be further confirmed by functional studies. Altogether, the interpretation of the pathogenic and likely pathogenic variants in the syndromic genes was credible based on the current evidence. However, with the accumulation of new knowledge regarding the correlation between genotype and genetic variants, our conclusions about pathogenicity could evolve.

In summary, targeted resequencing is an effective method for genetic testing in clinical settings, especially for polygenic hereditary diseases. Our resequencing panel can be used as an effective genetic testing method, supplementary to *FMR1* sequencing and CMA. The diagnostic yield of the panel reported in this study largely reflected the limited clinical interpretability of current high‐confidence ASD candidate genes, especially in the patient population in training centers, and can be expected to be higher in clinical patients. However, our testing panel can only be used to identify the genetic etiology of clinical patients who have obtained an accurate diagnosis of ASD. It cannot be used to screen referrals for suspected ASD in clinical settings or for the screening of the general population.

## CONFLICT OF INTERESTS

The authors declare that there are no conflict of interests.

## Supporting information

Supporting information

Supporting information

Supporting information

Supporting information
