# Supplementary material for: Targeted resequencing of 358 candidate genes for autism spectrum disorder in a Chinese cohort reveals diagnostic potential and genotype–phenotype correlations
Source: Hum Mutat. 2019 Apr 29;40(6):801–15. doi: 10.1002/humu.23724 (PMC6593842; doi:10.1002/humu.23724)
Supplement: Supplementary file 1 — Supporting information [file HUMU-40-801-s001.pdf]

Targeted resequencing of 358 candidate genes for autism spectrum disorder in a Chinese cohort reveals diagnostic potential and genotype-phenotype correlations

Wei-Zhen Zhou[1,2]*, Jie Zhang[1]*, Ziyi Li[1], Xiaojing Lin[3], Jiarui Li[1], Sheng Wang[3,4], Changhong Yang[3,5], Qixi Wu[6], Adam Yongxin Ye[1,7,8], Meng Wang[1], Dandan Wang[3], Tad Zhengzhang Pu[9], Yu-Yu Wu[10], Liping Wei[1]

[1]Center for Bioinformatics, State Key Laboratory of Protein and Plant Gene Research, School of Life Sciences, Peking University, Beijing, China
[2]State Key Laboratory of Cardiovascular Disease, Beijing Key Laboratory for Molecular Diagnostics of Cardiovascular Diseases, Diagnostic Laboratory Service, Fuwai Hospital, National Center for Cardiovascular Diseases, Chinese Academy of Medical Sciences and Peking Union Medical College, Beijing, China
[3]National Institute of Biological Sciences, Beijing, China
[4]College of Biological Sciences, China Agricultural University, Beijing, China
[5]College of Life Sciences, Beijing Normal University, Beijing, China
[6]School of Life Sciences, Peking University, Beijing, China
[7]Peking-Tsinghua Center for Life Sciences, Beijing, China
[8]Academy for Advanced Interdisciplinary Studies, Peking University, Beijing, China
[9]Shanghai United Family Hospital and Clinics, Shanghai, China
[10]Yuning Psychiatry Clinic, Taipei, Taiwan

Correspondence: Liping Wei, Center for Bioinformatics, State Key Laboratory of Protein and Plant Gene Research, School of Life Sciences, Peking University, Beijing, 100871, China. Email: weilp@mail.cbi.pku.edu.cn

*These authors contributed equally to this work.

Supp. Figure S1. Plot of the first two eigenvectors from EIGENSTRAT using 300 AIMs
Supp. Figure S2. 15q13.3 duplications confirmed by the Infinium GSAMD and CytoScan HD microarray platforms.
Supp. Table S1. Phenotypic assessments of ASD cases
Supp. Table S2. 111 syndromic ASD genes
Supp. Table S3. 247 non-syndromic ASD genes
Supp. Table S4. Target sequencing quality of 1,004 samples
Supp. Table S5. The distribution of the number of different variant sets for each gene
Supp. Table S6. The criteria of the ACMG-AMP guidelines used to determine variant classifications
Supp. Table S7. GSAMD and CytoScan HD results for 15q13.3 microduplications
Supp. Table S8. Phenotypes of carriers with pathogenic and likely pathogenic variants of syndromic genes and recurrent CNVs

Supp. Table S9. Phenotypes of carriers of *SHANK2* and *SHANK3* variants

Supp. Table S10. Phenotypes of carriers of 15q11-13 duplications

Supp. Table S11. Comparison of variants between cases with and without a specific phenotype

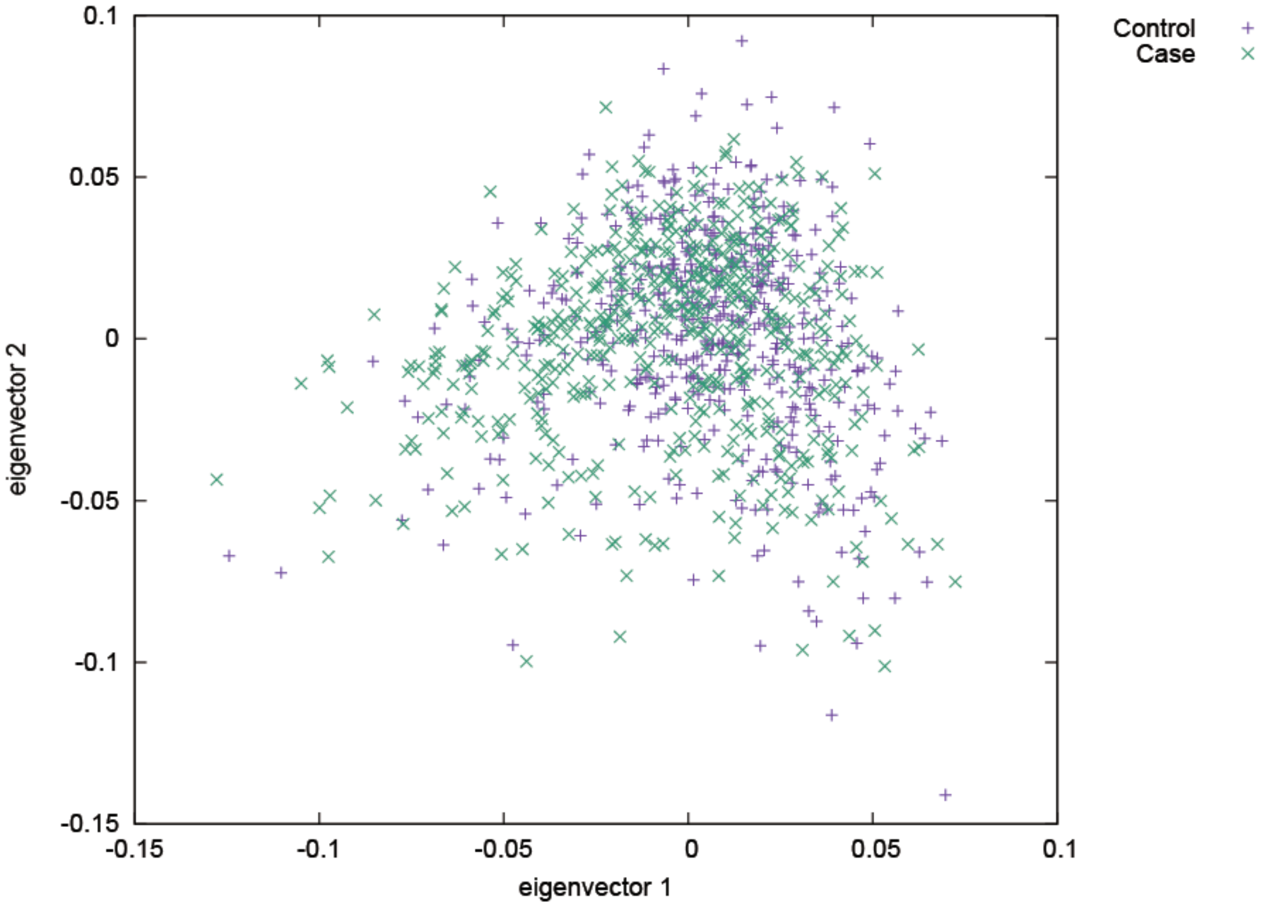

Supp. Figure S1. The first two eigenvectors from EIGENSTRAT using 300 Chinese AIMs are plotted. The statistical significance of the differences between 539 cases and 512 controls is 2.43×10^{-5}.

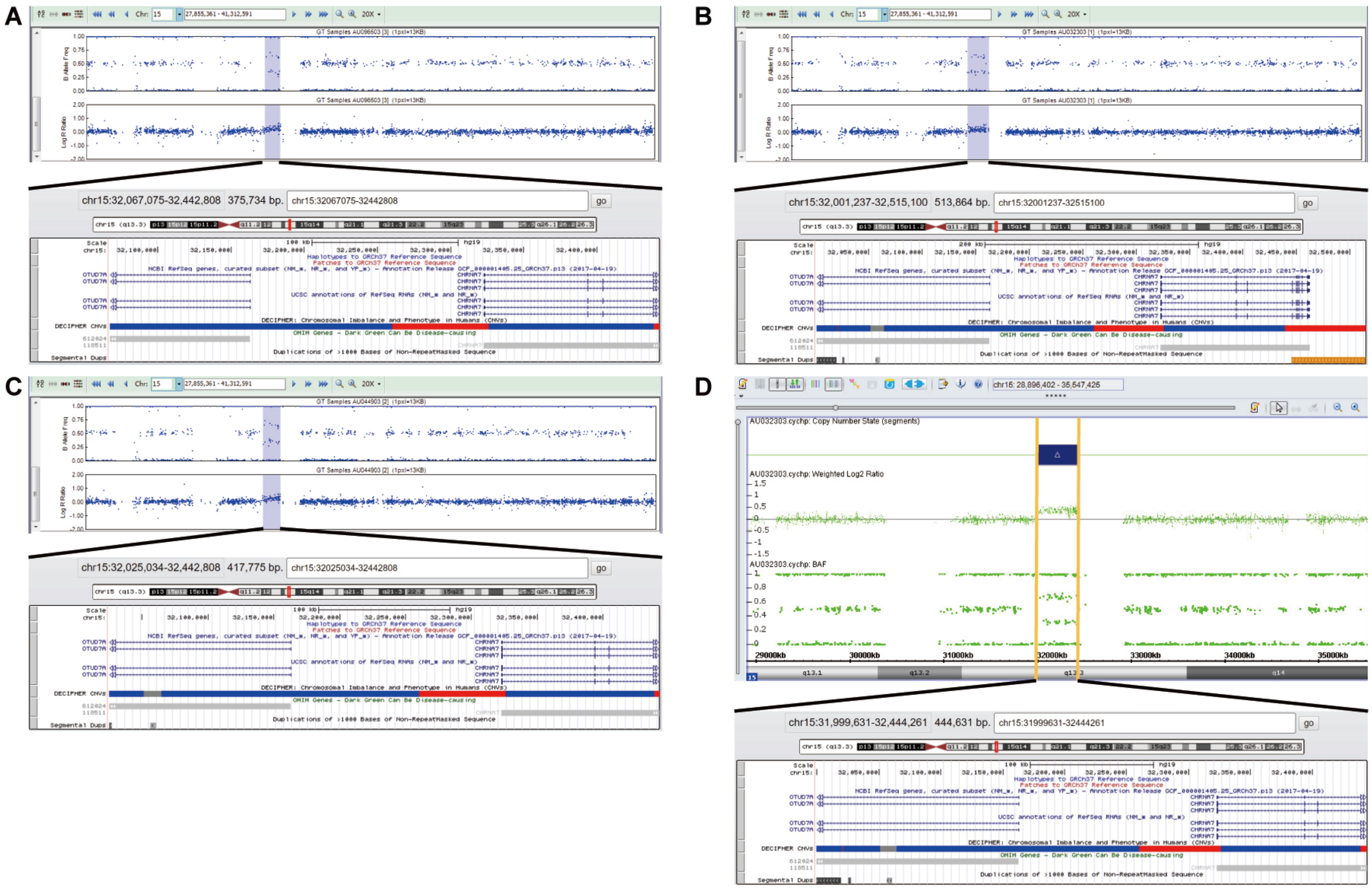

Supp. Figure S2. 15q13.3 duplications confirmed by the Infinium GSAMD and CytoScan HD microarray platforms. (A) A 375.73 kb duplication at 15q13.3 (chr15:32067075-32442808) in AU096503 confirmed by Infinium GSAMD; (B) A 513.86 kb duplication at 15q13.3 (chr15:32001237-32515100) in AU032303 confirmed by Infinium GSAMD; (C) A 417.77 kb duplication at 15q13.3 (chr15:32025034-32442808) in AU044903 confirmed by Infinium GSAMD; (D) A 444.63 kb duplication at 15q13.3 (chr15:33999631-32444261) in AU032303 confirmed by CytoScan HD. Log R ratio and B allele frequency are shown in the upper panel, and RefSeq gene annotations are displayed in the lower panel.

Supp. Table S1. Phenotypic assessments of ASD cases

Assessment/Scales	Proband/ Affected Child	Parents	Unaffected Sibling	Other Unaffected Relatives
Autism Diagnostic Interview-Revised (ADI-R)	√		√	
Autism Diagnostic Observation Scale (ADOS)	√			
Child Psychiatrist's Clinical Diagnosis	√			
Social Responsiveness Scale (SRS) - Parent report	√		√	
Repetitive Behavior Scale-Revised (RBS-R)	√			
Vineland Adaptive Behavior Scale (VABS)	√		√	
Combined Raven's Test	√			
Birth and Development History Interview	√			
Pedigree Information Form	√	√	√	√
Medical History Interview	√	√	√	√
Adult Autism Spectrum Quotient (AQ)		√		√
Broad Autism Phenotype Questionnaire (BAPQ)		√		√

Supp. Table S2. 111 syndromic ASD genes

Gene Symbol	Chr	Locus	Syndromes	Inheritance Pattern	Category
ACSL4	X	Xq22.3-q23	Mental retardation, X-linked 63	XL	Group 1
ADSL	22	22q13.1|22q13.2	Adenylosuccinate lyase deficiency	AR	Group 1
AFF2	X	Xq28	Fragile X mental retardation 2 (FRAXE)	XL	Group 1
AGTR2	X	Xq22-q23	Mental retardation, X-linked 88	XL	Group 1
AHI1	6	6q23.3	Leber congenital amaurosis	AR	Group 1
ALDH5A1	6	6p22	Succinic semialdehyde dehydrogenase deficiency	AR	Group 1
ALDH7A1	5	5q31	Pyridoxine-dependent epilepsy	AR	Group 1
ARHGEF6	X	Xq26.3	X-linked forms of mental retardation	XL	Group 1
ARX	X	Xp21	X-linked mental retardation	XL	Group 1
ATRX	X	Xq21.1	Alpha-thalassemia/mental retardation syndrome	XL	Group 1
BRAF	7	7q34	Cardio-facio-cutaneous syndrome	AD	Group 1
CACNA1F	X	Xp11.23	X-linked incomplete congenital stationary night blindness (CSNB2)	XL	Group 1

CDKL5	X	Xp22	Epileptic encephalopathy, early infantile, 2	XL	Group 1
CEP290	12	12q21.32	Joubert syndrome 5	AR	Group 1
CHD7	8	8q12.2	CHARGE syndrome	AD	Group 1
CREBBP	16	16p13.3	Rubinstein-Taybi syndrome	AD	Group 1
DCX	X	Xq22.3-q23	Type 1 lissencephaly	XL	Group 1
DMD	X	Xp21.2	Muscular dystrophy, Duchenne and Becker types	XL	Group 1
DMPK	19	19q13.3	Myotonic dystrophy 1 (Steinert disease)	AD	Group 1
EHMT1	9	9q34.3	9q subtelomeric deletion syndrome (Kleefstra syndrome)	AD	Group 1
FGD1	X	Xp11.21	Aarskog-Scott syndrome	XL	Group 1
FOXG1	14	14q13	Rett syndrome	AD	Group 1
FOXP1	3	3p14.1	Mental retardation with language impairment and autistic features	AD	Group 1
FTSJ1	X	Xp11.23	Mental retardation, X-linked-9	XL	Group 1
GAMT	19	19p13.3	Guanidine acetate methyltransferase (GAMT) deficiency	AR	Group 1
GRIA3	X	Xq25	Mental retardation, X-linked 94	XL	Group 1
HOXA1	7	7p15.3	Bosley-Salih-Alorainy syndrome	AR	Group 1
IGF2	11	11p15.5	Beckwith-Wiedermann syndrome	AD	Group 1
IL1RAPL1	X	Xp22.1-p21.3	X-linked mental retardation	XL	Group 1
IQSEC2	X	Xp11.22	Mental retardation, X-linked 1	XL	Group 1
KRAS	12	12p12.1	Cardio-facio-cutaneous syndrome	AD	Group 1
L1CAM	X	Xq28	MASA syndrome	XL	Group 1
MAP2K1	15	15q22.1-q22.33	Cardio-facio-cutaneous syndrome	AD	Group 1
MBD5	2	2q23.1	2q23.1 microdeletion syndrome	AD	Group 1
MED12	X	Xq13	Lujan-Fryns syndrome	XL	Group 1
MEF2C	5	5q14	5q14.3 microdeletion syndrome	AD	Group 1
MID1	X	Xp22	Opitz syndrome	XL	Group 1
MKKS	20	20p12	Bardet-Biedl syndrome	AR	Group 1
NDP	X	Xp11.4	Norrie disease	XL	Group 1
NF1	17	17q11.2	Neurofibromatosis type 1	AD	Group 1
NFIX	19	19p13.3	Sotos-like overgrowth syndrome	AD	Group 1
NHS	X	Xp22.13	Nance-Horan syndrome	XL	Group 1
NIPBL	5	5p13.2	Cornelia de Lange Syndrome	AD	Group 1
NLGN4X	X	Xp22.33	Mental retardation, X-linked	XL	Group 1

NRXN1	2	2p16.3	Pitt-Hopkins-like mental retardation	AR	Group 1
NSD1	5	5q35	Sotos syndrome	AD	Group 1
OCRL	X	Xq25	Lowe syndrome	XL	Group 1
OPHN1	X	Xq12	Mental retardation, X-linked, with cerebellar hypoplasia and distinctive facial appearance	XL	Group 1
PAFAH1B1	17	17p13.3	Lissencephaly 1	AD	Group 1
PAH	12	12q22-q24.2	Phenylketonuria	AR	Group 1
PCDH19	X	Xq13.3	Sporadic infantile epileptic encephalopathy	XL	Group 1
POMGNT1	1	1p34.1	Muscle-eye-brain disease (MEB)	AR	Group 1
PQBP1	X	Xp11.23	Renpenning syndrome	XL	Group 1
PTEN	10	10q23.3	PTEN hamartoma-tumor syndrome	AD	Group 1
PTPN11	12	12q24	Noonan syndrome	AD	Group 1
RAB39B	X	Xq28	Mental retardation, X-linked-72	XL	Group 1
RAI1	17	17p11.2	Smith-Magenis syndrome	AD	Group 1
RNF135	17	17q11.2	Overgrowth syndrome	AD	Group 1
RPE65	1	1p31	Leber congenital amaurosis	AR	Group 1
RPGRIP1L	16	16q12.2	COACH syndrome	AR	Group 1
SATB2	2	2q33	2q33.1 microdeletion syndrome	AD	Group 1
SCN1A	2	2q24.3	Dravet syndrome	AD	Group 1
SGSH	17	17q25.3	Sanfilippo syndrome A	AR	Group 1
SHANK3	22	22q13.3	22q13 deletion syndrome	AD	Group 1
SLC6A8	X	Xq28	creatine transporter deficiency	XL	Group 1
SLC9A6	X	Xq26.3	mental retardation, microcephaly, epilepsy, and ataxia	XL	Group 1
TBX1	22	22q11.21	22q11 deletion syndrome phenotype	AD	Group 1
UPF3B	X	Xq25-q26	Mental retardation, X-linked, syndromic 14	XL	Group 1
VPS13B	8	8q22.2	Cohen syndrome	AR	Group 1
YWHAE	17	17p13.3	Miller-Dieker syndrome	AD	Group 1
CACNA1C	12	12p13.3	Timothy syndrome	AD	Group 1
CNTNAP2	7	7q35	Pitt-Hopkins-like syndrome 1	AR	Group 1
DHCR7	11	11q13.4	Smith-Lemli-Opitz syndrome	AR	Group 1
FMR1	X	Xq27.3	fragile X syndrome	XL	Group 1
MECP2	X	Xq28	Rett syndrome	XL	Group 1

TSC1	9	9q34	Tuberous sclerosis-1	AD	Group 1
TSC2	16	16p13.3	Tuberous sclerosis-2	AD	Group 1
UBE3A	15	15q11.2	Angelman syndrome	AD	Group 1
AP1S2	X	Xp22.2	Mental retardation, X-linked 59	XL	Group 2
BTD	3	3p25	Biotinidase deficiency	AR	Group 2
CASK	X	Xp11.4	Mental retardation and microcephaly with pontine and cerebellar hypoplasia	XL	Group 2
DPYD	1	1p22	Dihydropyrimidine dehydrogenase deficiency	AR	Group 2
FGFR2	10	10q26	Apert syndrome	AD	Group 2
FOLR1	11	11q13.3-q14.1	Cerebral folate transport deficiency	AR	Group 2
GATM	15	15q21.1	Arginine:glycine amidinotransferase (AGAT) deficiency	AR	Group 2
GNS	12	12q14	Mucopolysaccharidosis type IIID (Sanfilippo disease D)	AR	Group 2
GRIN2B	12	12p12	autosomal dominant mental retardation	AD	Group 2
GUCY2D	17	17p13.1	Leber congenital amaurosis	AR	Group 2
HEPACAM	11	11q24.2	Megalencephalic leukoencephalopathy with subcortical cysts (recessive); leukodystrophy and macrocephaly (dominant)	AR/AD	Group 2
HGSNAT	8	8p11.21	Mucopolysaccharidosis type IIIC (Sanfilippo syndrome C)	AR	Group 2
HRAS	11	11p15.5	Costello syndrome	AD	Group 2
KCNJ11	11	11p15.1	DEND syndrome (developmental delay, epilepsy, and neonatal diabetes)	AD	Group 2
NEXMIF	X	Xq13.3	syndromic X-linked mental retardation	XL	Group 2
L2HGDH	14	14q21.3	L-2-hydroxyglutaric aciduria	AR	Group 2
LAMP2	X	Xq24	Danon disease	XL	Group 2
NAGLU	17	17q21	Mucopolysaccharidosis type IIIB (Sanfilippo syndrome B)	AR	Group 2
NPHP1	2	2q13	Joubert syndrome type 4, nephronophthisis	AR	Group 2
OTC	X	Xp21.1	Ornithine transcarbamylase deficiency	XL	Group 2
PHF6	X	Xq26.3	Borjeson-Forssman-Lehmann syndrome	XL	Group 2
PHF8	X	Xp11.22	Siderius-Hamel syndrome	XL	Group 2
POMT1	9	9q34.1	Limb-girdle muscular dystrophy with mental retardation; Walker-Warburg syndrome	AR	Group 2
PRSS12	4	4q28.1	autosomal recessive non-syndromic mental retardation	AR	Group 2
SMC1A	X	Xp11.22-p11.21	Cornelia de Lange syndrome	XL	Group 2
SYN1	X	Xp11.23	X-linked epilepsy and mental retardation	XL	Group 2
SYNGAP1	6	6p21.3	non-syndromic mental retardation	AD	Group 2

ZNF674	X	Xp11.3	non-syndromic X-linked mental retardation	XL	Group 2
ZNF81	X	Xp11.23	non-syndromic X-linked mental retardation	XL	Group 2
HDAC4	2	2q37.3	Brachydactyly mental retardation syndrome	AD	Group 3
CHRNA7	15	15q14	Microdeletion 15q13.3	AD	Group 3
TCF4	18	18q21.1	Pitt-Hopkins Syndrome	AD	Group 3
MAGEL2	15	15q11-q12	Prader-Willi syndrome	AD	Group 3

XL: X-linked, AD: autosomal dominant, AR: autosomal recessive

Supp. Table S3. 247 non-syndromic ASD genes

Gene Symbol	Chr	Locus	Level	Source
ADA	20	20q13.12	1	Association only
ADRB2	5	5q31-q32	1	Association only
ANK2	4	4q25-q27	1	Association and other
ANK3	10	10q21	1	Association and other
APOE	19	19q13.2	1	Association only
ARNT2	15	15q24	1	Association and other
ASMT	X|Y	Xp22.3|Yp11.3	1	Association and other
ATP10A	15	15q11.2	1	Association only
AVPR1A	12	12q14-q15	1	Association and other
BDNF	11	11p13	1	Association only
C4B	6	6p21.3	1	Association only
BICDL1	12	12q24.23	1	Association only
CDH10	5	5p14.2	1	Association only
CDH22	20	20q13.1	1	Association only
CDH9	5	5p14	1	Association only
CHD8	14	14q11.2	1	Association and other
COMT	22	22q11.21	1	Association only
CTNNA3	10	10q22.2	1	Association only
CUL3	2	2q36.2	1	Association and other
DISC1	1	1q42.1	1	Association and other
DLX6	7	7q22	1	Association and other
DNAH5	5	5p15.2	1	Association and other
DRD1	5	5q35.1	1	Association only
DRD2	11	11q23	1	Association only
DRD3	3	3q13.3	1	Association only
DUSP3	17	17q21	1	Association and other
DYRK1A	21	21q22.13	1	Association and other
EN2	7	7q36	1	Association only

ESRRB	14	14q24.3	1	Association only
FEZF1	7	7q31.32	1	Association only
FEZF2	3	3p14.2	1	Association and other
FHIT	3	3p14.2	1	Association only
FOXP2	7	7q31	1	Association and other
FRK	6	6q21-q22.3	1	Association only
GABRA4	4	4p12	1	Association only
GABRB3	15	15q11.2-q12	1	Association only
GALNT18	11	11p15.3	1	Association and other
GPR139	16	16p12.3	1	Association and other
GRIK2	6	6q16.3-q21	1	Association only
GRIN2A	16	16p13.2	1	Association only
GSE1	16	16q24.1	1	Association and other
HLA-DRB1	6	6p21.3	1	Association only
HS3ST5	6	6q21	1	Association only
IMMP2L	7	7q31	1	Association only
ITGA4	2	2q31.3	1	Association only
ITGB3	17	17q21.32	1	Association and other
KATNAL2	18	18q21.1	1	Association and other
KCNMA1	10	10q22.3	1	Association and other
KDM4C	9	9p24.1	1	Association only
KDM5C	X	Xp11.22-p11.21	1	Association and other
KIAA0100	17	17q11.2	1	Association and other
KIRREL3	11	11q24	1	Association and other
LAMB1	7	7q22	1	Association only
LRP1	12	12q13-q14	1	Association and other
LRRC1	6	6p12.1	1	Association only
LZTS2	10	10q24	1	Association only
MAOA	X	Xp11.3	1	Association and other
MBD1	18	18q21	1	Association and other

MEGF11	15	15q22.31	1	Association and other
MET	7	7q31	1	Association only
METTL22	16	16p13.2	1	Association only
MFSD6	2	2q32.2	1	Association only
MTF1	1	1p33	1	Association only
MYO16	13	13q33.3	1	Association only
MYO7B	2	2q21.1	1	Association and other
NLGN1	3	3q26.31	1	Association and other
NLGN3	X	Xq13.1	1	Association and other
NLGN4Y	Y	Yq11.221	1	Association and other
NRCAM	7	7q31	1	Association only
NTRK3	15	15q25	1	Association only
OMG	17	17q11.2	1	Association only
OXTR	3	3p25	1	Association only
PER1	17	17p13.1	1	Association and other
PITX1	5	5q31	1	Association only
PLXNA4	7	7q32.3	1	Association only
POGZ	1	1q21.3	1	Association and other
POU6F2	7	7p14.1	1	Association only
PRKCB	16	16p11.2	1	Association only
PRKX	X	Xp22.3	1	Association and other
RAPGEF4	2	2q31-q32	1	Association and other
RBFOX1	16	16p13.3	1	Association only
RELN	7	7q22	1	Association and other
RFX8	2	2q11.2	1	Association and other
RHOXF1	X	Xq24	1	Association only
RIMS1	6	6q12-q13	1	Association and other
RPL10	X	Xq28	1	Association and other
RPS6KA3	X	Xp22.2-p22.1	1	Association and other
SBF1	22	22q13.33	1	Association and other

SCN2A	2	2q24.3	1	Association and other
SEMA5A	5	5p15.2	1	Association only
SEZ6L2	16	16p11.2	1	Association and other
SHANK2	11	11q13.2	1	Association and other
SLC25A12	2	2q24	1	Association only
SLC30A5	5	5q12.1	1	Association and other
SLC6A4	17	17q11.2	1	Association and other
SLCO1C1	12	12p12.2	1	Association and other
ST8SIA2	15	15q26	1	Association only
STX1A	7	7q11.23	1	Association only
KMT5B	11	11q13.2	1	Association and other
SYPL1	7	7q22.3	1	Association only
TAS2R1	5	5p15	1	Association only
TBL1XR1	3	3q26.32	1	Association and other
TBR1	2	2q24	1	Association and other
TPH2	12	12q21.1	1	Association only
TRIO	5	5p15.2	1	Association and other
TSPAN12	7	7q31.31	1	Association only
TTN	2	2q31	1	Association and other
TUBA1A	12	12q13.12	1	Association and other
VASH1	14	14q24.3	1	Association only
WNT2	7	7q31.2	1	Association and other
XPO1	2	2p16	1	Association only
ZNF385B	2	2q31.2-q31.3	1	Association only
ARID1B	6	6q25.1	2	Association and other
AUTS2	7	7q11.22	2	Association and other
BCKDK	16	16p11.2	2	Association and other
CACNA1G	17	17q22	2	Association and other
CD38	4	4p15	2	Association only
CNTN4	3	3p26.3	2	Association and other

CTTNBP2	7	7q31	2	Association and other
DLGAP2	8	8p23	2	Association and other
DOCK4	7	7q31.1	2	Association only
EIF4E	4	4q21-q25	2	Association and other
EPHB2	1	1p36.1-p35	2	Association and other
FBXO33	14	14q21.1	2	Association only
GNB1L	22	22q11.2	2	Association and other
GRIN3B	19	19p13.3	2	Association and other
GSTM1	1	1p13.3	2	Association only
HTR3A	11	11q23.1	2	Association and other
JARID2	6	6p24-p23	2	Association only
LRFN5	14	14q21.1	2	Association only
LRP2	2	2q24-q31	2	Association and other
MACROD2	20	20p12.1	2	Association only
MAPK3	16	16p11.2	2	Association and other
MARK1	1	1q41	2	Association only
MRTFB	16	16p13.12	2	Association and other
MTHFR	1	1p36.3	2	Association only
NFIA	1	1p31.3-p31.2	2	Association and other
NTM	11	11q25	2	Association only
OTX1	2	2p13	2	Association only
PAX6	11	11p13	2	Association and other
PTCHD1	X	Xp22.11	2	Association and other
SLC13A1	7	7q31-q32	2	Association only
SLTM	15	15q22.1	2	Association only
SND1	7	7q31.3	2	Association and other
SNRPN	15	15q11.2	2	Association only
SYT17	16	16p12.3	2	Association only
UPP2	2	2q24.1	2	Association only
BRCA2	13	13q12.3	2	Association and other

CDH8	16	16q22.1	2	Association and other
CSTF2T	10	10q11	2	Association and other
DAB1	1	1p32-p31	2	Association only
DNER	2	2q36.3	2	Association only
EP400	12	12q24.33	2	Association and other
ERBB4	2	2q33.3-q34	2	Association only
ESR1	6	6q25.1	2	Association only
FAT1	4	4q35	2	Association and other
GABRB1	4	4p12	2	Association only
ADGRV1	5	5q13	2	Association and other
GRM8	7	7q31.3-q32.1	2	Association only
HLA-A	6	6p21.3	2	Association only
HTR1B	6	6q13	2	Association only
JMJD1C	10	10q21.3	2	Association and other
KCND2	7	7q31	2	Association only
LAMC3	9	9q31-q34	2	Association and other
NTNG1	1	1p13.3	2	Association and other
PRKN	6	6q25.2-q27	2	Association and other
PTS	11	11q22.3	2	Association only
RIMS3	1	1p34.2	2	Association and other
SPAST	2	2p24-p21	2	Association and other
STXBP1	9	9q34.1	2	Association and other
TAS2R3	7	7q31.3-q32	2	Association and other
TM4SF19	3	3q29	2	Association and other
TMLHE	X	Xq28	2	Association and other
TRIP12	2	2q36.3	2	Association and other
ABCA13	7	7p12.3	3	Association and other
BANK1	4	4q24	3	Association and other
CCDC138	2	2q12.3	3	Association and other
COL25A1	4	4q25	3	Association and other

DEAF1	11	11p15.5	3	Association and other
DNMT3A	2	2p23	3	Association and other
DSTYK	1	1q32.1	3	Association and other
FAM91A1	8	8q24.13	3	Association and other
LMCD1	3	3p26-p24	3	Association and other
MED13L	12	12q24.21	3	Association and other
MICALCL	11	11p15.3	3	Association and other
MTHFS	15	15q25.1	3	Association and other
NR3C2	4	4q31.1	3	Association and other
PARP10	8	8q24.3	3	Association and other
PIWIL4	11	11q21	3	Association and other
PPM1D	17	17q23.2	3	Association and other
PRIM2	6	6p12-p11.1	3	Association and other
PRPF39	14	14q21.2	3	Association and other
RAB2A	8	8q12.1	3	Association and other
RNF38	9	9p13	3	Association and other
S100G	X	Xp22.2	3	Association and other
WDR55	5	5q31.3	3	Association and other
ZNF493	19	19p12	3	Association and other
ACTR3C	7	7q36.1	3	Association and other
AGXT2	5	5p13	3	Association and other
AMT	3	3p21.2-p21.1	3	Association and other
ARSF	X	Xp22.3	3	Association and other
ARSH	X	Xp22.33	3	Association and other
ATP1B4	X	Xq24	3	Association and other
BEND2	X	Xp22.13	3	Association and other
CFHR2	1	1q31.3	3	Association and other
CT45A5	X	Xq26.3	3	Association and other
CXCR3	X	Xq13	3	Association and other
CYP2C18	10	10q24	3	Association and other

DBF4B	17	17q21.31|17q21	3	Association and other
DGAT2L6	X	Xq13.1	3	Association and other
DRP2	X	Xq22	3	Association and other
GPHN	14	14q23.3	3	Association and other
ADGRG4	X	Xq26.3	3	Association and other
GYG2	X	Xp22.3	3	Association and other
HAP1	17	17q21.2-q21.3	3	Association and other
HAUS7	X	Xq28	3	Association and other
ITIH6	X	Xp11.22-p11.21	3	Association and other
KIAA1210	X	Xq24	3	Association and other
MFSD4B	6	6q22	3	Association and other
KNG1	3	3q27	3	Association and other
LRRC29	16	16q22.1	3	Association and other
LRRC69	8	8q21.3	3	Association and other
LUZP4	X	Xq23	3	Association and other
MAGEC3	X	Xq27.2	3	Association and other
MCF2	X	Xq27	3	Association and other
MICB	6	6p21.3	3	Association and other
MTMR8	X	Xq11.2	3	Association and other
OTOGL	12	12q21.31	3	Association and other
PCDH11X	X	Xq21.3	3	Association and other
PEX7	6	6q23.3	3	Association and other
PIR	X	Xp22.2	3	Association and other
PLAT	8	8p12	3	Association and other
PRDX4	X	Xp22.11	3	Association and other
PTH2R	2	2q33	3	Association and other
PZP	12	12p13-p12.2	3	Association and other
RNF128	X	Xq22.3	3	Association and other
SLC22A14	3	3p21.3	3	Association and other
SRPX2	X	Xq21.33-q23	3	Association and other

SYN2	3	3p25	3	Association and other
SYNE1	6	6q25	3	Association and other
TGM4	3	3p22-p21.33	3	Association and other
USH2A	1	1q41	3	Association and other
VSIG1	X	Xq22.3	3	Association and other
YWHAZ	8	8q23.1	3	Association and other
ZCCHC13	X	Xq13.2	3	Association and other
RTL4	X	Xq23	3	Association and other
ZNF157	X	Xp11.2	3	Association and other

Supp. Table S6 The criteria of ACMG-AMP guidelines used to determine variant classifications

Sample	cDNA	Protein	Genotype	Gene	Type	Origin	Inheritance Pattern	Classification	ACMG classification	ACMG-AMP criteria
AU076603	c.1229delC	p.Pro410fs	het	*IQSEC2*	fs del	de novo or father	XLD	LP	P	PVS1, PM2, PP4
AU065903	c.766C>T	p.Arg256*	het	*MEF2C*	stopgain	de novo	AD	P	P	PVS1, PS2, PM2, PP3, PP4, PP5
AU049703	c.403-1G>T	-	het	*MEF2C*	splicing	de novo	AD	P	P	PVS1, PS2, PM2, PP3, PP4
AU012204	c.973C>T	p.Arg325*	het	*MBD5*	stopgain	de novo	AD	P	P	PVS1, PS2, PM2, PP3, PP4
AU060803	c.404dupG	p.Gly136fs	het	*PTEN*	fs ins	father	AD	LP	P	PVS1, PM2, PP4, BS2
AU037503	c.460dupC	p.Arg154fs	het	*PTEN*	fs ins	mother	AD	LP	P	PVS1, PM2, PP4, BS2
AU095803	c.2854C>T	p.Arg952*	hom	*CDKL5*	stopgain	mother	XLD	LP	Likely Benign	PVS1, BS2, BP4, BP6
AU048503	c.803+1G>A	-	het	*HEPACAM*	splicing	father	AD/AR	LP	VUS	PM2, PP3, PP4, BS2
AU065403	c.1742dupT	p.Leu581fs	het	*NF1*	fs ins	mother	AD	LP	VUS	PVS1, PP4, BS2
AU065503	c.1742dupT	p.Leu581fs	het	*NF1*	fs ins	mother	AD	LP	VUS	PVS1, BS2
AU099703	c.1742dupT	p.Leu581fs	het	*NF1*	fs ins	mother	AD	LP	VUS	PVS1, BS2
AU052603	c.1015delG	p.Val339fs	het	*RNF135*	fs del	father	AD	P	VUS	PP5, BS2
AU095503	c.1015delG	p.Val339fs	het	*RNF135*	fs del	father	AD	P	VUS	PP5, BS2
AU056603	c.3424_3425del	p.Leu1142fs	het	*SHANK3*	fs del	de novo	AD	P	P	PVS1, PS2, PM2, PP4
AU013503	c.3679dupG	p.Ala1227fs	het	*SHANK3*	fs ins	de novo or father	AD	P	P	PVS1, PP4, PP5, BS1
AU035703	c.3679dupG	p.Ala1227fs	het	*SHANK3*	fs ins	de novo	AD	P	P	PVS1, PS2, PP4, PP5, BS1
AU039303	c.4753_4763del	p.Lys1585fs	het	*TSC2*	fs del	de novo	AD	P	P	PVS1, PS2, PM2, PP4
AU018703	c.199G>A	p.Asp67Asn	het	*MAP2K1*	missense	de novo or father	AD	P	P	PS1, PM1, PM2, PP2, PP3, PP4, PP5

AU017403	c.1081C>G	p.Leu361Val	het	*TSC2*	missense	mother	AD	LP	LP	PM1, PM2, PP2, PP3, PP5, BS2

fs del: frameshift deletion, fs ins: frameshift insertion

XLD: X-linked dominant, AD: autosomal dominant, AR: autosomal recessive

P: Pathogenic, LP: Likely pathogenic, VUS: Variants of uncertain significance

Supp. Table S7. GSAMD and CytoScan HD results for 15q13.3 microduplications

Sample	Position	Size (kbp)	Band	Type	Confidence	# Markers
**GSAMD**						
AU096503	15:32067075-32442808	375.73	15q13.3	dup	238.38	88
AU032303	15:32001237-32515100	513.86	15q13.3	dup	278.32	100
AU044903	15:32025034-32442808	417.77	15q13.3	dup	326.50	96
**CytoScan HD**						
AU032303	15:33999631-32444261	444.63	15q13.3	dup		577

Supp. Table S8. Phenotypes of carriers with pathogenic and likely pathogenic variants of syndromic genes and recurrent CNVs

Sample	Syndrome	Previously Reported Clinical Characteristics	Clinical Symptoms of the Carrier
AU076603	Mental retardation, X-linked 1/78	Mild to severe intellectual disability, and some had seizures, language delay, and nonspecific facial dysmorphism (females were much less severely affected)	Moderate intellectual disability, severe language delay (spoke her first word at 7 years old) and lack phrase speech, delayed psychomotor development, abnormal EEG with too much slow wave activity, and her father also had a language delay
AU065903	Chromosome 5q14.3 deletion syndrome	Mental retardation, absent speech, seizures, poor eye contact, and stereotypic movements, some had hypotonia, delayed motor development, and variable brain anomalies on imaging	Mental retardation, febrile seizures (onset at around age 1 year), lack of speech, poor eye contact, restricted or stereotyped interests and activities, poor motor coordination and abnormal gait, delay of motor development, abnormal MRI, and sleep problems

AU049703	Chromosome 5q14.3 deletion syndrome	Mental retardation, absent speech, seizures, poor eye contact, and stereotypic movements, some had hypotonia, delayed motor development, variable dysmorphic features, and variable brain anomalies on imaging	Mental retardation, epilepsy, lack of speech, poor eye contact, restricted or stereotyped interests and activities, poor motor coordination and abnormal gait, motor development delay, having sleep problems; his mother also had epilepsy, and he has a family history of schizophrenia, cerebral palsy, febrile seizure, and undiagnosed development disabilities.
AU012204	Mental retardation	Mental retardation, developmental delay, motor delay, severe language impairment, and autistic-like behavioral problems, some have seizures, hypotonia, feeding difficulties, sleep disturbances, short stature, craniofacial abnormalities	Mental retardation, infantile hypotonia, global developmental delay (walked independently at age 22 months and said his first word and phrase at age 37 months), sleep disturbances, short stature (-2.8SD), congenital malformations (funnel chest, delayed closure of fontanel), abnormal imaging (scalp atrophy), normal hearing and vision, and no seizure
AU060803	PTEN hamartoma-tumor syndrome	Macrocephaly	Abnormal head shape, delayed closure of fontanel, ribs valgus
AU037503	PTEN hamartoma-tumor syndrome	Macrocephaly	Large head circumference during pregnancy
AU048503	Megalencephalic leukoencephalopathy with subcortical cysts (recessive); leukodystrophy and macrocephaly (dominant)	Infantile-onset of macrocephaly, mildly delayed motor development, some have mild residual hypotonia or clumsiness, and mental retardation	Development delay (walked independently at age 15 months, spoke her first word at age 41 months and can only say less than 5 words), mental retardation and head circumference data is unavailable
AU065403	Neurofibromatosis, type 1	Cafe-au-lait spots, Lisch nodules in the eye, fibromatous tumors of the skin, some children have learning, behavioral, and vision problems, scoliosis, macrocephaly, short stature, pseudarthrosis, difficulties with coordination, and a mild form of epilepsy	Development delay (walked independently at age 14 months, spoke his first word at age 45 months and his first phrase at age 47 months), vision problem (amblyopia), abnormal EEG
AU065503	Neurofibromatosis, type 1	Cafe-au-lait spots, Lisch nodules in the eye, fibromatous tumors of the skin, some children have learning, behavioral, and vision problems, scoliosis, macrocephaly, short stature, pseudarthrosis, difficulties with coordination, and a mild form of epilepsy	Development delay, feeding difficulties, skeletal abnormality: leg bowing, early closure of cranial suture, skin, tooth and ear abnormalities, difficulty with balance and movement coordination, hyperactive, compulsive and aggressive behaviors, and sleep problems

AU099703	Neurofibromatosis, type 1	Cafe-au-lait spots, Lisch nodules in the eye, fibromatous tumors of the skin, some children have learning, behavioral, and vision problems, scoliosis, macrocephaly, short stature, pseudarthrosis, difficulties with coordination, and a mild form of epilepsy	Development delay (walked independently at age 16 months, spoke his first phrase at age 36 months), abnormal gait, poor movement coordination, hypotonia
AU052603	Overgrowth syndrome	Increased postnatal height and weight, macrocephaly, learning disability, dysmorphic facial features	Language delay, febrile seizures, hyperactive, delayed closure of fontanel, abnormal gait, and sleep problems
AU095503	Overgrowth syndrome	Increased postnatal height and weight, macrocephaly, learning disability, dysmorphic facial features	Language delay, hyperactive, and sleep problems
AU056603	Chromosome 22q13.3 deletion syndrome	Severe verbal and social deficits	Mental retardation, global developmental delay (walked independently at age 18 months, spoke her first word at age 40 months, and can only say less than 5 words), severe social deficits and typical autistic behavior problems, strabismus, inverted eyelashes, late teething, poor motor coordination and abnormal gait, and sleep problems
AU013503	Chromosome 22q13.3 deletion syndrome		Severe social deficits, spoke her first word at age 45 months and first phase at age 48 months; her father also suffered from language delay
AU035703	Chromosome 22q13.3 deletion syndrome		Severe social deficits, spoke her first word and phase at age 24 months, but lost language skills at 42 months, abnormal gait, hyperactive, and sleep problems
AU039303	Tuberous sclerosis-2	Tuberous sclerosis, epilepsy, intellectual disturbance, and sebaceous adenoma	Tuberous sclerosis and epilepsy
AU018703	Cardio-facio-cutaneous syndrome-3	Characteristic craniofacial features, cardiac anomalies, hair and skin abnormalities, postnatal growth deficiency, hypotonia, and developmental delay	Congenital heart disease, development delay (walked independently at age 20 months), and sleep problems
AU017403	Tuberous sclerosis-2	Tuberous sclerosis, epilepsy, intellectual disturbance, and sebaceous adenoma	Abnormal MRI (abnormal left frontal lobe), masturbation syndrome
AU077403	15q11-13 duplication syndrome	Moderate to severe intellectual disability, ataxia, hypotonia, epilepsy, developmental delays,	A four-year-old male with autism, harboring a 15q11.2-12 duplication, has low muscle tone and his muscles feel soft. He displayed problems in motor

		language is impaired or absent in most, no dysmorphic features, and congenital malformations are rare	coordination, especially gross motor movement such as walking unstably; as well as displayed developmental delay; intellectual disability; and impaired language. His standard score of the adaptive behavior composite evaluated by Vineland Adaptive Behavior Scales (VABS) is 60.
AU096503			A two-year-old male with ASD; he carries the 15q13.3 duplication. He has a family history of mental retardation and showed developmental delay and is language impaired.
AU032303			A four-year-old male with typical autism, harboring a 15q13.3 duplication, has a family history of developmental delay and almost no language and intellectual deficiency. VABS's standard score is 55. In addition, he has rough skin and decreased sensitivity to pain.
AU044903			A five-year-old male with autism; he also carries a 15q13.3 duplication. He has a family history of language delay and epilepsy and showed poor motor coordination and abnormal gait. He has speech and language delay and deficits, and was noted to be hyperactive.
AU042703	2q37 deletion syndrome	Mild facial dysmorphism, vascular and skeletal malformations, a variable degree of intellectual disability, and hypotonia	A three-year-old girl with autism carrying a 2q37 deletion. She also suffered from rickets, which affects the development of bones, and hernia. She has a family history of language delay and epilepsy and exhibited a general developmental delay and mental retardation.
AU033603	22q11.2 deletion syndrome	Variable symptoms	A three-year-old girl with autism has a *de novo* 22q11.2 deletion. She exhibited ear malformation, loss of hearing in the left ear, dental anomalies, strabismus, and amblyopia. She has a severe milk allergy, serious feeding problems, no language, cognitive deficit, and developmental delay (walking independently by 36 months). While lacking the symptoms of congenital heart disease and palatal abnormalities, we found many features that were previously reported and consistent with this syndrome's phenotype.

Supp. Table S9. Phenotypes of carriers of *SHANK2* and *SHANK3* variants

**Sample ID**	**AU056603**	**AU013503**	**AU035703**	**AU067003**	**AU102003**	**AU074003**
**Variant**	*SHANK3* c.3424_3425del (p.Leu1142fs)	*SHANK3* c.3679dupG (p.Ala1227fs)	*SHANK3* c.3679dupG (p.Ala1227fs)	*SHANK3* c.593C>G (p.Ala198Gly)	*SHANK3* c.898C>T (p.Pro300Ser)	*SHANK2* c.2540_2541del (p.Ser847*)
**Gender**	Female	Female	Female	Male	Male	Male
**Age**	3y6m	4y7m	5y4m	5y1m	5y4m	4y
**Socialization domain of VABS (mental age)**	NA	NA	2m	4m	6m	4m
**Motor development delay**	−	−	−	−	−	−
**Language development delay**	+	+	+	+	+	+
**Unusual sensory interests**	−	−	+	−	−	+
**Hyper-responsivity to sensory stimuli**	−	−	−	+	−	+
**Macrocephaly**	−	NA	−	−	−	−
**Microcephaly**	−	NA	−	−	−	−
**Abnormal EEG**	NA	−	NA	NA	NA	−
**Abnormal MRI**	NA	−	−	NA	NA	NA
**Skills loss**	−	−	+	−	−	−
**Epilepsy**	−	−	−	−	−	−
**Sleep disorder**	+	−	+	+	+	+
**Gastrointestinal problems**	+	−	+	−	−	−
**Hypotonia**	−	−	−	−	−	−
**Hypertonia**	−	−	−	−	−	−
**Hyperactivity**	−	−	+	+	+	−
**Anxiety**	−	−	−	−	−	−

**Aggressive behavior**	−	−	+	−	−	−
**Obsessive behavior**	−	−	−	−	+	−
**Feeding difficulty**	−	−	−	−	−	−
**Abnormal gait**	+	−	+	+	+	−
**Other problems**	Inverted eyelashes, strabismus	Decayed tooth, myopia and astigmatism	−	−	−	−

Supp. Table S10. Phenotypes of carriers of 15q11-13 duplications

**Sample ID**	**AU077403**	**AU096503**	**AU032303**	**AU044903**
**Variant**	15q11.2-12	15q13.3	15q13.3	15q13.3
**Gender**	Male	Male	Male	Male
**Age**	4y5m	2y9m	4y6m	5y2m
**Mental retardation**	+	NA	+	NA
**Socialization domain of VABS (mental age)**	4m	NA	2m	NA
**Motor development delay**	+	−	−	−
**Language development delay**	+	+	+	+
**Unusual sensory interests**	+	−	+	+
**Hyper-responsivity to sensory stimuli**	+	−	−	+
**Macrocephaly**	−	−	−	−
**Microcephaly**	−	−	−	−
**Abnormal EEG**	−	−	NA	NA
**Abnormal MRI**	−	−	NA	NA
**Skills loss**	−	−	−	−
**Epilepsy**	−	−	−	−
**Sleep disorder**	−	−	−	−
**Gastrointestinal problems**	+	−	−	−
**Hypotonia**	+	−	−	−
**Hypertonia**	−	−	−	−
**Hyperactivity**	−	−	+	+

**Anxiety**	−	−	−	−
**Aggressive behavior**	−	+	+	−
**Obsessive behavior**	−	−	+	+
**Feeding difficulty**	−	−	−	−
**Abnormal gait**	−	−	−	+
**Poor coordination of movements**	+	−	−	+
**Other problems**	Suspected skeletal abnormality	−	Rough skin	Suspected joint abnormality

Supp. Table S11. Comparison of variants between cases with and without a specific phenotype

**Phenotype**	**# Cases with specific phenotype**	**# Cases without specific phenotype**	**Unadjusted *p* value**	**Adjusted *p* value**
**Loss of language skills**	43	471	0.091	0.73
**Minimally verbal**	113	330	0.093	0.75
**Unusual sensory interests**	49	205	0.22	1
**Self-injurious behavior**	13	449	0.34	1
**Epilepsy/Tics**	31	432	**0.017**	**0.13**
**Gastrointestinal problems**	76	386	0.36	1
**Hypotonia**	22	420	0.67	1
**Insensitivity to pain**	120	304	0.27	1
