# Supplementary material for: Targeted resequencing of 358 candidate genes for autism spectrum disorder in a Chinese cohort reveals diagnostic potential and genotype–phenotype correlations
Source: Hum Mutat. 2019 Apr 29;40(6):801–15. doi: 10.1002/humu.23724 (PMC6593842; doi:10.1002/humu.23724)
Supplement: Supplementary file 2 — Supporting information [file HUMU-40-801-s002.docx]

**Targeted resequencing of 358 candidate genes for autism spectrum disorder in a Chinese cohort**

**reveals diagnostic potential and genotype-phenotype correlations**

Wei-Zhen Zhou^1,2^*, Jie Zhang^1^*, Ziyi Li^1^, Xiaojing Lin^3^, Jiarui Li^1^, Sheng Wang^3,4^, Changhong Yang^3,5^, Qixi Wu^6^, Adam Yongxin Ye^1,7,8^, Meng Wang^1^, Dandan Wang^3^, Tad Zhengzhang Pu^9^, Yu-Yu Wu^10^, Liping Wei^1^

^1^Center for Bioinformatics, State Key Laboratory of Protein and Plant Gene Research, School of Life Sciences, Peking University, Beijing, China

^2^State Key Laboratory of Cardiovascular Disease, Beijing Key Laboratory for Molecular Diagnostics of Cardiovascular Diseases, Diagnostic Laboratory Service, Fuwai Hospital, National Center for Cardiovascular Diseases, Chinese Academy of Medical Sciences and Peking Union Medical College, Beijing, China

^3^National Institute of Biological Sciences, Beijing, China

^4^College of Biological Sciences, China Agricultural University, Beijing, China

^5^College of Life Sciences, Beijing Normal University, Beijing, China

^6^School of Life Sciences, Peking University, Beijing, China

^7^Peking-Tsinghua Center for Life Sciences, Beijing, China

^8^Academy for Advanced Interdisciplinary Studies, Peking University, Beijing, China

^9^Shanghai United Family Hospital and Clinics, Shanghai, China

^10^Yuning Psychiatry Clinic, Taipei, Taiwan

Correspondence: Liping Wei, Center for Bioinformatics, State Key Laboratory of Protein and Plant Gene Research, School of Life Sciences, Peking University, Beijing, 100871, China. Email: weilp@mail.cbi.pku.edu.cn

*These authors contributed equally to this work.

**Supp. Figure S1.** Plot of the first two eigenvectors from EIGENSTRAT using 300 AIMs

**Supp. Figure S2.** 15q13.3 duplications confirmed by the Infinium GSAMD and CytoScan HD microarray platforms.

**Supp. Table S1.** Phenotypic assessments of ASD cases

**Supp. Table S2.** 111 syndromic ASD genes

**Supp. Table S3.** 247 non-syndromic ASD genes

**Supp. Table S4.** Target sequencing quality of 1,004 samples

**Supp. Table S5.** The distribution of the number of different variant sets for each gene

**Supp. Table S6.** The criteria of the ACMG-AMP guidelines used to determine variant classifications

**Supp. Table S7.** GSAMD and CytoScan HD results for 15q13.3 microduplications

**Supp. Table S8.** Phenotypes of carriers with pathogenic and likely pathogenic variants of syndromic genes and recurrent CNVs

**Supp. Table S9.** Phenotypes of carriers of *SHANK2* and *SHANK3* variants

**Supp. Table S10.** Phenotypes of carriers of 15q11-13 duplications

**Supp. Table S11.** Comparison of variants between cases with and without a specific phenotype


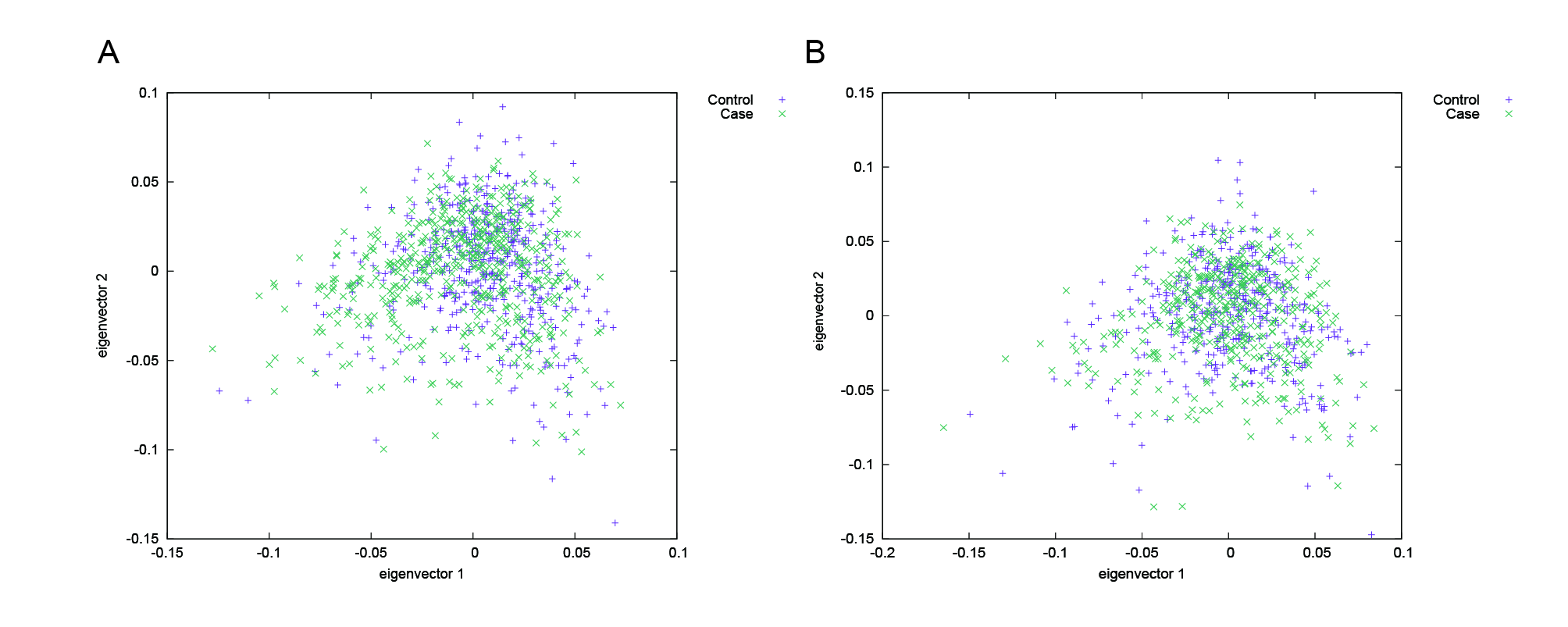


# Supp. Figure S1. The first two eigenvectors from EIGENSTRAT using 300 Chinese AIMs are plotted. The statistical significance of the differences between 539 cases and 512 controls is 2.43×10^-5^.

**
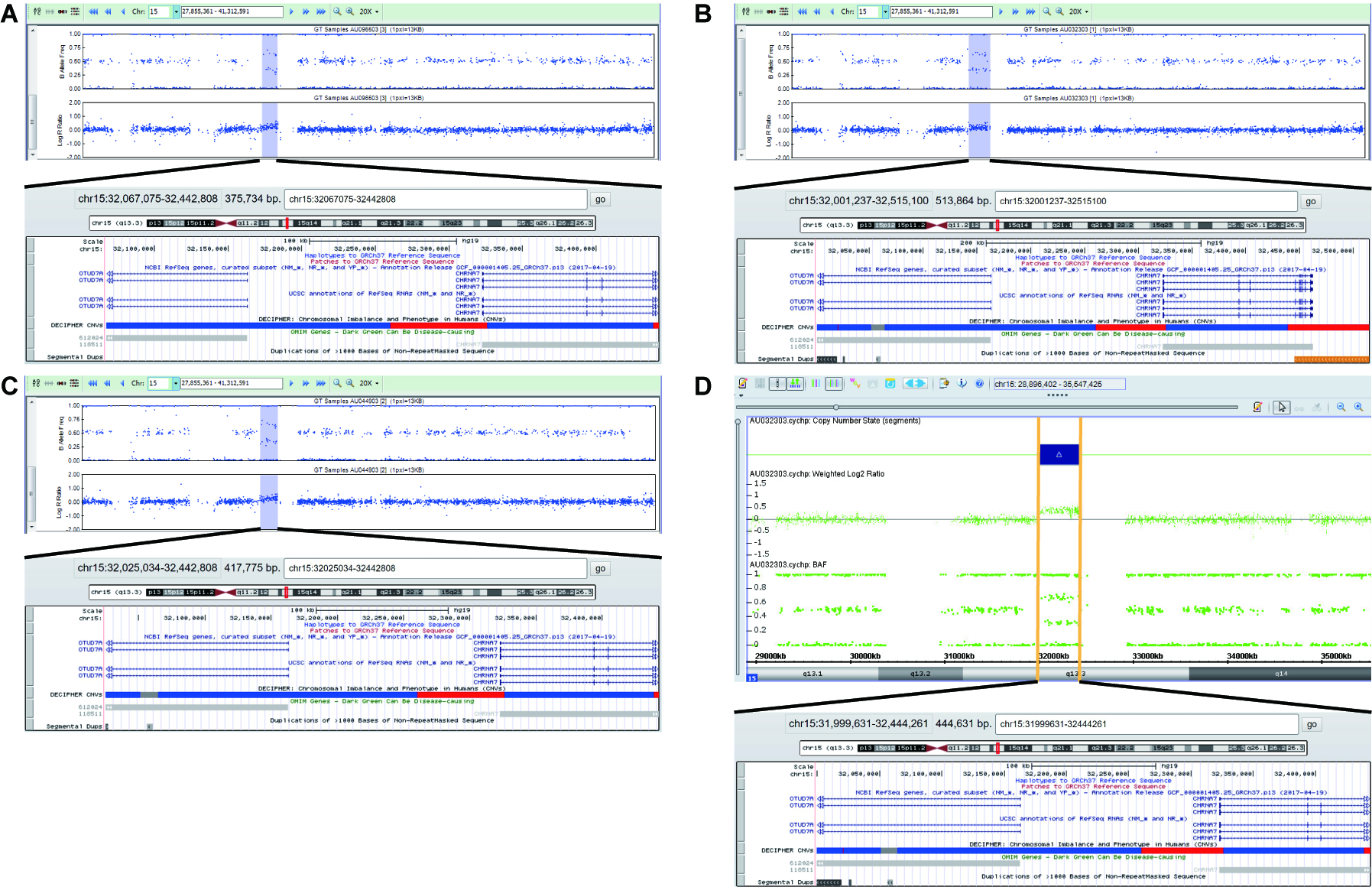
**

**Supp. Figure S2. 15q13.3 duplications confirmed by** **the I****nfinium** **GSAMD and** **CytoScan HD microarray platforms.** (A) A 375.73 kb duplication at 15q13.3 (chr15:32067075-32442808) in AU096503 confirmed by Infinium GSAMD; (B) A 513.86 kb duplication at 15q13.3 (chr15:32001237-32515100) in AU032303 confirmed by Infinium GSAMD; (C) A 417.77 kb duplication at 15q13.3 (chr15:32025034-32442808) in AU044903 confirmed by Infinium GSAMD; (D) A 444.63 kb duplication at 15q13.3 (chr15:33999631-32444261) in AU032303 confirmed by CytoScan HD. Log R ratio and B allele frequency are shown in the upper panel, and RefSeq gene annotations are displayed in the lower panel.

**Supp. Table S1.** Phenotypic assessments of ASD cases

| **Assessment/Scales** | **Proband/ Affected Child** | **Parents** | **Unaffected Sibling** | **Other Unaffected Relatives** |
| --- | --- | --- | --- | --- |
| Autism Diagnostic Interview-Revised (ADI-R) | √ |  | √ |  |
| Autism Diagnostic Observation Scale (ADOS) | √ |  |  |  |
| Child Psychiatrist’s Clinical Diagnosis | √ |  |  |  |
| Social Responsiveness Scale (SRS) - Parent report | √ |  | √ |  |
| Repetitive Behavior Scale-Revised (RBS-R) | √ |  |  |  |
| Vineland Adaptive Behavior Scale (VABS) | √ |  | √ |  |
| Combined Raven’s Test | √ |  |  |  |
| Birth and Development History Interview | √ |  |  |  |
| Pedigree Information Form | √ | √ | √ | √ |
| Medical History Interview | √ | √ | √ | √ |
| Adult Autism Spectrum Quotient (AQ) |  | √ |  | √ |
| Broad Autism Phenotype Questionnaire (BAPQ) |  | √ |  | √ |

**Supp. Table S2.** 111 syndromic ASD genes

| **Gene Symbol** | **Chr** | **Locus** | **Syndromes** | **Inheritance Pattern** | **Category** |
| --- | --- | --- | --- | --- | --- |
| *ACSL4* | X | Xq22.3-q23 | Mental retardation, X-linked 63 | XL | Group 1 |
| *ADSL* | 22 | 22q13.1\|22q13.2 | Adenylosuccinate lyase deficiency | AR | Group 1 |
| *AFF2* | X | Xq28 | Fragile X mental retardation 2 (FRAXE) | XL | Group 1 |
| *AGTR2* | X | Xq22-q23 | Mental retardation, X-linked 88 | XL | Group 1 |
| *AHI1* | 6 | 6q23.3 | Leber congenital amaurosis | AR | Group 1 |
| *ALDH5A1* | 6 | 6p22 | Succinic semialdehyde dehydrogenase deficiency | AR | Group 1 |
| *ALDH7A1* | 5 | 5q31 | Pyridoxine-dependent epilepsy | AR | Group 1 |
| *ARHGEF6* | X | Xq26.3 | X-linked forms of mental retardation | XL | Group 1 |
| *ARX* | X | Xp21 | X-linked mental retardation | XL | Group 1 |
| *ATRX* | X | Xq21.1 | Alpha-thalassemia/mental retardation syndrome | XL | Group 1 |
| *BRAF* | 7 | 7q34 | Cardio-facio-cutaneous syndrome | AD | Group 1 |
| *CACNA1F* | X | Xp11.23 | X-linked incomplete congenital stationary night blindness (CSNB2) | XL | Group 1 |
| *CDKL5* | X | Xp22 | Epileptic encephalopathy, early infantile, 2 | XL | Group 1 |
| *CEP290* | 12 | 12q21.32 | Joubert syndrome 5 | AR | Group 1 |
| *CHD7* | 8 | 8q12.2 | CHARGE syndrome | AD | Group 1 |
| *CREBBP* | 16 | 16p13.3 | Rubinstein-Taybi syndrome | AD | Group 1 |
| *DCX* | X | Xq22.3-q23 | Type 1 lissencephaly | XL | Group 1 |
| *DMD* | X | Xp21.2 | Muscular dystrophy, Duchenne and Becker types | XL | Group 1 |
| *DMPK* | 19 | 19q13.3 | Myotonic dystrophy 1 (Steinert disease) | AD | Group 1 |
| *EHMT1* | 9 | 9q34.3 | 9q subtelomeric deletion syndrome (Kleefstra syndrome) | AD | Group 1 |
| *FGD1* | X | Xp11.21 | Aarskog-Scott syndrome | XL | Group 1 |
| *FOXG1* | 14 | 14q13 | Rett syndrome | AD | Group 1 |
| *FOXP1* | 3 | 3p14.1 | Mental retardation with language impairment and autistic features | AD | Group 1 |
| *FTSJ1* | X | Xp11.23 | Mental retardation, X-linked-9 | XL | Group 1 |
| *GAMT* | 19 | 19p13.3 | Guanidine acetate methyltransferase (GAMT) deficiency | AR | Group 1 |
| *GRIA3* | X | Xq25 | Mental retardation, X-linked 94 | XL | Group 1 |
| *HOXA1* | 7 | 7p15.3 | Bosley-Salih-Alorainy syndrome | AR | Group 1 |
| *IGF2* | 11 | 11p15.5 | Beckwith-Wiedermann syndrome | AD | Group 1 |
| *IL1RAPL1* | X | Xp22.1-p21.3 | X-linked mental retardation | XL | Group 1 |
| *IQSEC2* | X | Xp11.22 | Mental retardation, X-linked 1 | XL | Group 1 |
| *KRAS* | 12 | 12p12.1 | Cardio-facio-cutaneous syndrome | AD | Group 1 |
| *L1CAM* | X | Xq28 | MASA syndrome | XL | Group 1 |
| *MAP2K1* | 15 | 15q22.1-q22.33 | Cardio-facio-cutaneous syndrome | AD | Group 1 |
| *MBD5* | 2 | 2q23.1 | 2q23.1 microdeletion syndrome | AD | Group 1 |
| *MED12* | X | Xq13 | Lujan-Fryns syndrome | XL | Group 1 |
| *MEF2C* | 5 | 5q14 | 5q14.3 microdeletion syndrome | AD | Group 1 |
| *MID1* | X | Xp22 | Opitz syndrome | XL | Group 1 |
| *MKKS* | 20 | 20p12 | Bardet-Biedl syndrome | AR | Group 1 |
| *NDP* | X | Xp11.4 | Norrie disease | XL | Group 1 |
| *NF1* | 17 | 17q11.2 | Neurofibromatosis type 1 | AD | Group 1 |
| *NFIX* | 19 | 19p13.3 | Sotos-like overgrowth syndrome | AD | Group 1 |
| *NHS* | X | Xp22.13 | Nance-Horan syndrome | XL | Group 1 |
| *NIPBL* | 5 | 5p13.2 | Cornelia de Lange Syndrome | AD | Group 1 |
| *NLGN4X* | X | Xp22.33 | Mental retardation, X-linked | XL | Group 1 |
| *NRXN1* | 2 | 2p16.3 | Pitt-Hopkins-like mental retardation | AR | Group 1 |
| *NSD1* | 5 | 5q35 | Sotos syndrome | AD | Group 1 |
| *OCRL* | X | Xq25 | Lowe syndrome | XL | Group 1 |
| *OPHN1* | X | Xq12 | Mental retardation, X-linked, with cerebellar hypoplasia and distinctive facial appearance | XL | Group 1 |
| *PAFAH1B1* | 17 | 17p13.3 | Lissencephaly 1 | AD | Group 1 |
| *PAH* | 12 | 12q22-q24.2 | Phenylketonuria | AR | Group 1 |
| *PCDH19* | X | Xq13.3 | Sporadic infantile epileptic encephalopathy | XL | Group 1 |
| *POMGNT1* | 1 | 1p34.1 | Muscle-eye-brain disease (MEB) | AR | Group 1 |
| *PQBP1* | X | Xp11.23 | Renpenning syndrome | XL | Group 1 |
| *PTEN* | 10 | 10q23.3 | PTEN hamartoma-tumor syndrome | AD | Group 1 |
| *PTPN11* | 12 | 12q24 | Noonan syndrome | AD | Group 1 |
| *RAB39B* | X | Xq28 | Mental retardation, X-linked-72 | XL | Group 1 |
| *RAI1* | 17 | 17p11.2 | Smith-Magenis syndrome | AD | Group 1 |
| *RNF135* | 17 | 17q11.2 | Overgrowth syndrome | AD | Group 1 |
| *RPE65* | 1 | 1p31 | Leber congenital amaurosis | AR | Group 1 |
| *RPGRIP1L* | 16 | 16q12.2 | COACH syndrome | AR | Group 1 |
| *SATB2* | 2 | 2q33 | 2q33.1 microdeletion syndrome | AD | Group 1 |
| *SCN1A* | 2 | 2q24.3 | Dravet syndrome | AD | Group 1 |
| *SGSH* | 17 | 17q25.3 | Sanfilippo syndrome A | AR | Group 1 |
| *SHANK3* | 22 | 22q13.3 | 22q13 deletion syndrome | AD | Group 1 |
| *SLC6A8* | X | Xq28 | creatine transporter deficiency | XL | Group 1 |
| *SLC9A6* | X | Xq26.3 | mental retardation, microcephaly, epilepsy, and ataxia | XL | Group 1 |
| *TBX1* | 22 | 22q11.21 | 22q11 deletion syndrome phenotype | AD | Group 1 |
| *UPF3B* | X | Xq25-q26 | Mental retardation, X-linked, syndromic 14 | XL | Group 1 |
| *VPS13B* | 8 | 8q22.2 | Cohen syndrome | AR | Group 1 |
| *YWHAE* | 17 | 17p13.3 | Miller-Dieker syndrome | AD | Group 1 |
| *CACNA1C* | 12 | 12p13.3 | Timothy syndrome | AD | Group 1 |
| *CNTNAP2* | 7 | 7q35 | Pitt-Hopkins-like syndrome 1 | AR | Group 1 |
| *DHCR7* | 11 | 11q13.4 | Smith-Lemli-Opitz syndrome | AR | Group 1 |
| *FMR1* | X | Xq27.3 | fragile X syndrome | XL | Group 1 |
| *MECP2* | X | Xq28 | Rett syndrome | XL | Group 1 |
| *TSC1* | 9 | 9q34 | Tuberous sclerosis-1 | AD | Group 1 |
| *TSC2* | 16 | 16p13.3 | Tuberous sclerosis-2 | AD | Group 1 |
| *UBE3A* | 15 | 15q11.2 | Angelman syndrome | AD | Group 1 |
| *AP1S2* | X | Xp22.2 | Mental retardation, X-linked 59 | XL | Group 2 |
| *BTD* | 3 | 3p25 | Biotinidase deficiency | AR | Group 2 |
| *CASK* | X | Xp11.4 | Mental retardation and microcephaly with pontine and cerebellar hypoplasia | XL | Group 2 |
| *DPYD* | 1 | 1p22 | Dihydropyrimidine dehydrogenase deficiency | AR | Group 2 |
| *FGFR2* | 10 | 10q26 | Apert syndrome | AD | Group 2 |
| *FOLR1* | 11 | 11q13.3-q14.1 | Cerebral folate transport deficiency | AR | Group 2 |
| *GATM* | 15 | 15q21.1 | Arginine:glycine amidinotransferase (AGAT) deficiency | AR | Group 2 |
| *GNS* | 12 | 12q14 | Mucopolysaccharidosis type IIID (Sanfilippo disease D) | AR | Group 2 |
| *GRIN2B* | 12 | 12p12 | autosomal dominant mental retardation | AD | Group 2 |
| *GUCY2D* | 17 | 17p13.1 | Leber congenital amaurosis | AR | Group 2 |
| *HEPACAM* | 11 | 11q24.2 | Megalencephalic leukoencephalopathy with subcortical cysts (recessive); leukodystrophy and macrocephaly (dominant) | AR/AD | Group 2 |
| *HGSNAT* | 8 | 8p11.21 | Mucopolysaccharidosis type IIIC (Sanfilippo syndrome C) | AR | Group 2 |
| *HRAS* | 11 | 11p15.5 | Costello syndrome | AD | Group 2 |
| *KCNJ11* | 11 | 11p15.1 | DEND syndrome (developmental delay, epilepsy, and neonatal diabetes) | AD | Group 2 |
| *NEXMIF* | X | Xq13.3 | syndromic X-linked mental retardation | XL | Group 2 |
| *L2HGDH* | 14 | 14q21.3 | L-2-hydroxyglutaric aciduria | AR | Group 2 |
| *LAMP2* | X | Xq24 | Danon disease | XL | Group 2 |
| *NAGLU* | 17 | 17q21 | Mucopolysaccharidosis type IIIB (Sanfilippo syndrome B) | AR | Group 2 |
| *NPHP1* | 2 | 2q13 | Joubert syndrome type 4, nephronophthisis | AR | Group 2 |
| *OTC* | X | Xp21.1 | Ornithine transcarbamylase deficiency | XL | Group 2 |
| *PHF6* | X | Xq26.3 | Borjeson-Forssman-Lehmann syndrome | XL | Group 2 |
| *PHF8* | X | Xp11.22 | Siderius-Hamel syndrome | XL | Group 2 |
| *POMT1* | 9 | 9q34.1 | Limb-girdle muscular dystrophy with mental retardation; Walker-Warburg syndrome | AR | Group 2 |
| *PRSS12* | 4 | 4q28.1 | autosomal recessive non-syndromic mental retardation | AR | Group 2 |
| *SMC1A* | X | Xp11.22-p11.21 | Cornelia de Lange syndrome | XL | Group 2 |
| *SYN1* | X | Xp11.23 | X-linked epilepsy and mental retardation | XL | Group 2 |
| *SYNGAP1* | 6 | 6p21.3 | non-syndromic mental retardation | AD | Group 2 |
| *ZNF674* | X | Xp11.3 | non-syndromic X-linked mental retardation | XL | Group 2 |
| *ZNF81* | X | Xp11.23 | non-syndromic X-linked mental retardation | XL | Group 2 |
| *HDAC4* | 2 | 2q37.3 | Brachydactyly mental retardation syndrome | AD | Group 3 |
| *CHRNA7* | 15 | 15q14 | Microdeletion 15q13.3 | AD | Group 3 |
| *TCF4* | 18 | 18q21.1 | Pitt-Hopkins Syndrome | AD | Group 3 |
| *MAGEL2* | 15 | 15q11-q12 | Prader-Willi syndrome | AD | Group 3 |

XL: X-linked, AD: autosomal dominant, AR: autosomal recessive

**Supp. Table S3.** 247 non-syndromic ASD genes

| **Gene Symbol** | **Chr** | **Locus** | **Level** | **Source** |
| --- | --- | --- | --- | --- |
| *ADA* | 20 | 20q13.12 | 1 | Association only |
| *ADRB2* | 5 | 5q31-q32 | 1 | Association only |
| *ANK2* | 4 | 4q25-q27 | 1 | Association and other |
| *ANK3* | 10 | 10q21 | 1 | Association and other |
| *APOE* | 19 | 19q13.2 | 1 | Association only |
| *ARNT2* | 15 | 15q24 | 1 | Association and other |
| *ASMT* | X\|Y | Xp22.3\|Yp11.3 | 1 | Association and other |
| *ATP10A* | 15 | 15q11.2 | 1 | Association only |
| *AVPR1A* | 12 | 12q14-q15 | 1 | Association and other |
| *BDNF* | 11 | 11p13 | 1 | Association only |
| *C4B* | 6 | 6p21.3 | 1 | Association only |
| *BICDL1* | 12 | 12q24.23 | 1 | Association only |
| *CDH10* | 5 | 5p14.2 | 1 | Association only |
| *CDH22* | 20 | 20q13.1 | 1 | Association only |
| *CDH9* | 5 | 5p14 | 1 | Association only |
| *CHD8* | 14 | 14q11.2 | 1 | Association and other |
| *COMT* | 22 | 22q11.21 | 1 | Association only |
| *CTNNA3* | 10 | 10q22.2 | 1 | Association only |
| *CUL3* | 2 | 2q36.2 | 1 | Association and other |
| *DISC1* | 1 | 1q42.1 | 1 | Association and other |
| *DLX6* | 7 | 7q22 | 1 | Association and other |
| *DNAH5* | 5 | 5p15.2 | 1 | Association and other |
| *DRD1* | 5 | 5q35.1 | 1 | Association only |
| *DRD2* | 11 | 11q23 | 1 | Association only |
| *DRD3* | 3 | 3q13.3 | 1 | Association only |
| *DUSP3* | 17 | 17q21 | 1 | Association and other |
| *DYRK1A* | 21 | 21q22.13 | 1 | Association and other |
| *EN2* | 7 | 7q36 | 1 | Association only |
| *ESRRB* | 14 | 14q24.3 | 1 | Association only |
| *FEZF1* | 7 | 7q31.32 | 1 | Association only |
| *FEZF2* | 3 | 3p14.2 | 1 | Association and other |
| *FHIT* | 3 | 3p14.2 | 1 | Association only |
| *FOXP2* | 7 | 7q31 | 1 | Association and other |
| *FRK* | 6 | 6q21-q22.3 | 1 | Association only |
| *GABRA4* | 4 | 4p12 | 1 | Association only |
| *GABRB3* | 15 | 15q11.2-q12 | 1 | Association only |
| *GALNT18* | 11 | 11p15.3 | 1 | Association and other |
| *GPR139* | 16 | 16p12.3 | 1 | Association and other |
| *GRIK2* | 6 | 6q16.3-q21 | 1 | Association only |
| *GRIN2A* | 16 | 16p13.2 | 1 | Association only |
| *GSE1* | 16 | 16q24.1 | 1 | Association and other |
| *HLA-DRB1* | 6 | 6p21.3 | 1 | Association only |
| *HS3ST5* | 6 | 6q21 | 1 | Association only |
| *IMMP2L* | 7 | 7q31 | 1 | Association only |
| *ITGA4* | 2 | 2q31.3 | 1 | Association only |
| *ITGB3* | 17 | 17q21.32 | 1 | Association and other |
| *KATNAL2* | 18 | 18q21.1 | 1 | Association and other |
| *KCNMA1* | 10 | 10q22.3 | 1 | Association and other |
| *KDM4C* | 9 | 9p24.1 | 1 | Association only |
| *KDM5C* | X | Xp11.22-p11.21 | 1 | Association and other |
| *KIAA0100* | 17 | 17q11.2 | 1 | Association and other |
| *KIRREL3* | 11 | 11q24 | 1 | Association and other |
| *LAMB1* | 7 | 7q22 | 1 | Association only |
| *LRP1* | 12 | 12q13-q14 | 1 | Association and other |
| *LRRC1* | 6 | 6p12.1 | 1 | Association only |
| *LZTS2* | 10 | 10q24 | 1 | Association only |
| *MAOA* | X | Xp11.3 | 1 | Association and other |
| *MBD1* | 18 | 18q21 | 1 | Association and other |
| *MEGF11* | 15 | 15q22.31 | 1 | Association and other |
| *MET* | 7 | 7q31 | 1 | Association only |
| *METTL22* | 16 | 16p13.2 | 1 | Association only |
| *MFSD6* | 2 | 2q32.2 | 1 | Association only |
| *MTF1* | 1 | 1p33 | 1 | Association only |
| *MYO16* | 13 | 13q33.3 | 1 | Association only |
| *MYO7B* | 2 | 2q21.1 | 1 | Association and other |
| *NLGN1* | 3 | 3q26.31 | 1 | Association and other |
| *NLGN3* | X | Xq13.1 | 1 | Association and other |
| *NLGN4Y* | Y | Yq11.221 | 1 | Association and other |
| *NRCAM* | 7 | 7q31 | 1 | Association only |
| *NTRK3* | 15 | 15q25 | 1 | Association only |
| *OMG* | 17 | 17q11.2 | 1 | Association only |
| *OXTR* | 3 | 3p25 | 1 | Association only |
| *PER1* | 17 | 17p13.1 | 1 | Association and other |
| *PITX1* | 5 | 5q31 | 1 | Association only |
| *PLXNA4* | 7 | 7q32.3 | 1 | Association only |
| *POGZ* | 1 | 1q21.3 | 1 | Association and other |
| *POU6F2* | 7 | 7p14.1 | 1 | Association only |
| *PRKCB* | 16 | 16p11.2 | 1 | Association only |
| *PRKX* | X | Xp22.3 | 1 | Association and other |
| *RAPGEF4* | 2 | 2q31-q32 | 1 | Association and other |
| *RBFOX1* | 16 | 16p13.3 | 1 | Association only |
| *RELN* | 7 | 7q22 | 1 | Association and other |
| *RFX8* | 2 | 2q11.2 | 1 | Association and other |
| *RHOXF1* | X | Xq24 | 1 | Association only |
| *RIMS1* | 6 | 6q12-q13 | 1 | Association and other |
| *RPL10* | X | Xq28 | 1 | Association and other |
| *RPS6KA3* | X | Xp22.2-p22.1 | 1 | Association and other |
| *SBF1* | 22 | 22q13.33 | 1 | Association and other |
| *SCN2A* | 2 | 2q24.3 | 1 | Association and other |
| *SEMA5A* | 5 | 5p15.2 | 1 | Association only |
| *SEZ6L2* | 16 | 16p11.2 | 1 | Association and other |
| *SHANK2* | 11 | 11q13.2 | 1 | Association and other |
| *SLC25A12* | 2 | 2q24 | 1 | Association only |
| *SLC30A5* | 5 | 5q12.1 | 1 | Association and other |
| *SLC6A4* | 17 | 17q11.2 | 1 | Association and other |
| *SLCO1C1* | 12 | 12p12.2 | 1 | Association and other |
| *ST8SIA2* | 15 | 15q26 | 1 | Association only |
| *STX1A* | 7 | 7q11.23 | 1 | Association only |
| *KMT5B* | 11 | 11q13.2 | 1 | Association and other |
| *SYPL1* | 7 | 7q22.3 | 1 | Association only |
| *TAS2R1* | 5 | 5p15 | 1 | Association only |
| *TBL1XR1* | 3 | 3q26.32 | 1 | Association and other |
| *TBR1* | 2 | 2q24 | 1 | Association and other |
| *TPH2* | 12 | 12q21.1 | 1 | Association only |
| *TRIO* | 5 | 5p15.2 | 1 | Association and other |
| *TSPAN12* | 7 | 7q31.31 | 1 | Association only |
| *TTN* | 2 | 2q31 | 1 | Association and other |
| *TUBA1A* | 12 | 12q13.12 | 1 | Association and other |
| *VASH1* | 14 | 14q24.3 | 1 | Association only |
| *WNT2* | 7 | 7q31.2 | 1 | Association and other |
| *XPO1* | 2 | 2p16 | 1 | Association only |
| *ZNF385B* | 2 | 2q31.2-q31.3 | 1 | Association only |
| *ARID1B* | 6 | 6q25.1 | 2 | Association and other |
| *AUTS2* | 7 | 7q11.22 | 2 | Association and other |
| *BCKDK* | 16 | 16p11.2 | 2 | Association and other |
| *CACNA1G* | 17 | 17q22 | 2 | Association and other |
| *CD38* | 4 | 4p15 | 2 | Association only |
| *CNTN4* | 3 | 3p26.3 | 2 | Association and other |
| *CTTNBP2* | 7 | 7q31 | 2 | Association and other |
| *DLGAP2* | 8 | 8p23 | 2 | Association and other |
| *DOCK4* | 7 | 7q31.1 | 2 | Association only |
| *EIF4E* | 4 | 4q21-q25 | 2 | Association and other |
| *EPHB2* | 1 | 1p36.1-p35 | 2 | Association and other |
| *FBXO33* | 14 | 14q21.1 | 2 | Association only |
| *GNB1L* | 22 | 22q11.2 | 2 | Association and other |
| *GRIN3B* | 19 | 19p13.3 | 2 | Association and other |
| *GSTM1* | 1 | 1p13.3 | 2 | Association only |
| *HTR3A* | 11 | 11q23.1 | 2 | Association and other |
| *JARID2* | 6 | 6p24-p23 | 2 | Association only |
| *LRFN5* | 14 | 14q21.1 | 2 | Association only |
| *LRP2* | 2 | 2q24-q31 | 2 | Association and other |
| *MACROD2* | 20 | 20p12.1 | 2 | Association only |
| *MAPK3* | 16 | 16p11.2 | 2 | Association and other |
| *MARK1* | 1 | 1q41 | 2 | Association only |
| *MRTFB* | 16 | 16p13.12 | 2 | Association and other |
| *MTHFR* | 1 | 1p36.3 | 2 | Association only |
| *NFIA* | 1 | 1p31.3-p31.2 | 2 | Association and other |
| *NTM* | 11 | 11q25 | 2 | Association only |
| *OTX1* | 2 | 2p13 | 2 | Association only |
| *PAX6* | 11 | 11p13 | 2 | Association and other |
| *PTCHD1* | X | Xp22.11 | 2 | Association and other |
| *SLC13A1* | 7 | 7q31-q32 | 2 | Association only |
| *SLTM* | 15 | 15q22.1 | 2 | Association only |
| *SND1* | 7 | 7q31.3 | 2 | Association and other |
| *SNRPN* | 15 | 15q11.2 | 2 | Association only |
| *SYT17* | 16 | 16p12.3 | 2 | Association only |
| *UPP2* | 2 | 2q24.1 | 2 | Association only |
| *BRCA2* | 13 | 13q12.3 | 2 | Association and other |
| *CDH8* | 16 | 16q22.1 | 2 | Association and other |
| *CSTF2T* | 10 | 10q11 | 2 | Association and other |
| *DAB1* | 1 | 1p32-p31 | 2 | Association only |
| *DNER* | 2 | 2q36.3 | 2 | Association only |
| *EP400* | 12 | 12q24.33 | 2 | Association and other |
| *ERBB4* | 2 | 2q33.3-q34 | 2 | Association only |
| *ESR1* | 6 | 6q25.1 | 2 | Association only |
| *FAT1* | 4 | 4q35 | 2 | Association and other |
| *GABRB1* | 4 | 4p12 | 2 | Association only |
| *ADGRV1* | 5 | 5q13 | 2 | Association and other |
| *GRM8* | 7 | 7q31.3-q32.1 | 2 | Association only |
| *HLA-A* | 6 | 6p21.3 | 2 | Association only |
| *HTR1B* | 6 | 6q13 | 2 | Association only |
| *JMJD1C* | 10 | 10q21.3 | 2 | Association and other |
| *KCND2* | 7 | 7q31 | 2 | Association only |
| *LAMC3* | 9 | 9q31-q34 | 2 | Association and other |
| *NTNG1* | 1 | 1p13.3 | 2 | Association and other |
| *PRKN* | 6 | 6q25.2-q27 | 2 | Association and other |
| *PTS* | 11 | 11q22.3 | 2 | Association only |
| *RIMS3* | 1 | 1p34.2 | 2 | Association and other |
| *SPAST* | 2 | 2p24-p21 | 2 | Association and other |
| *STXBP1* | 9 | 9q34.1 | 2 | Association and other |
| *TAS2R3* | 7 | 7q31.3-q32 | 2 | Association and other |
| *TM4SF19* | 3 | 3q29 | 2 | Association and other |
| *TMLHE* | X | Xq28 | 2 | Association and other |
| *TRIP12* | 2 | 2q36.3 | 2 | Association and other |
| *ABCA13* | 7 | 7p12.3 | 3 | Association and other |
| *BANK1* | 4 | 4q24 | 3 | Association and other |
| *CCDC138* | 2 | 2q12.3 | 3 | Association and other |
| *COL25A1* | 4 | 4q25 | 3 | Association and other |
| *DEAF1* | 11 | 11p15.5 | 3 | Association and other |
| *DNMT3A* | 2 | 2p23 | 3 | Association and other |
| *DSTYK* | 1 | 1q32.1 | 3 | Association and other |
| *FAM91A1* | 8 | 8q24.13 | 3 | Association and other |
| *LMCD1* | 3 | 3p26-p24 | 3 | Association and other |
| *MED13L* | 12 | 12q24.21 | 3 | Association and other |
| *MICALCL* | 11 | 11p15.3 | 3 | Association and other |
| *MTHFS* | 15 | 15q25.1 | 3 | Association and other |
| *NR3C2* | 4 | 4q31.1 | 3 | Association and other |
| *PARP10* | 8 | 8q24.3 | 3 | Association and other |
| *PIWIL4* | 11 | 11q21 | 3 | Association and other |
| *PPM1D* | 17 | 17q23.2 | 3 | Association and other |
| *PRIM2* | 6 | 6p12-p11.1 | 3 | Association and other |
| *PRPF39* | 14 | 14q21.2 | 3 | Association and other |
| *RAB2A* | 8 | 8q12.1 | 3 | Association and other |
| *RNF38* | 9 | 9p13 | 3 | Association and other |
| *S100G* | X | Xp22.2 | 3 | Association and other |
| *WDR55* | 5 | 5q31.3 | 3 | Association and other |
| *ZNF493* | 19 | 19p12 | 3 | Association and other |
| *ACTR3C* | 7 | 7q36.1 | 3 | Association and other |
| *AGXT2* | 5 | 5p13 | 3 | Association and other |
| *AMT* | 3 | 3p21.2-p21.1 | 3 | Association and other |
| *ARSF* | X | Xp22.3 | 3 | Association and other |
| *ARSH* | X | Xp22.33 | 3 | Association and other |
| *ATP1B4* | X | Xq24 | 3 | Association and other |
| *BEND2* | X | Xp22.13 | 3 | Association and other |
| *CFHR2* | 1 | 1q31.3 | 3 | Association and other |
| *CT45A5* | X | Xq26.3 | 3 | Association and other |
| *CXCR3* | X | Xq13 | 3 | Association and other |
| *CYP2C18* | 10 | 10q24 | 3 | Association and other |
| *DBF4B* | 17 | 17q21.31\|17q21 | 3 | Association and other |
| *DGAT2L6* | X | Xq13.1 | 3 | Association and other |
| *DRP2* | X | Xq22 | 3 | Association and other |
| *GPHN* | 14 | 14q23.3 | 3 | Association and other |
| *ADGRG4* | X | Xq26.3 | 3 | Association and other |
| *GYG2* | X | Xp22.3 | 3 | Association and other |
| *HAP1* | 17 | 17q21.2-q21.3 | 3 | Association and other |
| *HAUS7* | X | Xq28 | 3 | Association and other |
| *ITIH6* | X | Xp11.22-p11.21 | 3 | Association and other |
| *KIAA1210* | X | Xq24 | 3 | Association and other |
| *MFSD4B* | 6 | 6q22 | 3 | Association and other |
| *KNG1* | 3 | 3q27 | 3 | Association and other |
| *LRRC29* | 16 | 16q22.1 | 3 | Association and other |
| *LRRC69* | 8 | 8q21.3 | 3 | Association and other |
| *LUZP4* | X | Xq23 | 3 | Association and other |
| *MAGEC3* | X | Xq27.2 | 3 | Association and other |
| *MCF2* | X | Xq27 | 3 | Association and other |
| *MICB* | 6 | 6p21.3 | 3 | Association and other |
| *MTMR8* | X | Xq11.2 | 3 | Association and other |
| *OTOGL* | 12 | 12q21.31 | 3 | Association and other |
| *PCDH11X* | X | Xq21.3 | 3 | Association and other |
| *PEX7* | 6 | 6q23.3 | 3 | Association and other |
| *PIR* | X | Xp22.2 | 3 | Association and other |
| *PLAT* | 8 | 8p12 | 3 | Association and other |
| *PRDX4* | X | Xp22.11 | 3 | Association and other |
| *PTH2R* | 2 | 2q33 | 3 | Association and other |
| *PZP* | 12 | 12p13-p12.2 | 3 | Association and other |
| *RNF128* | X | Xq22.3 | 3 | Association and other |
| *SLC22A14* | 3 | 3p21.3 | 3 | Association and other |
| *SRPX2* | X | Xq21.33-q23 | 3 | Association and other |
| *SYN2* | 3 | 3p25 | 3 | Association and other |
| *SYNE1* | 6 | 6q25 | 3 | Association and other |
| *TGM4* | 3 | 3p22-p21.33 | 3 | Association and other |
| *USH2A* | 1 | 1q41 | 3 | Association and other |
| *VSIG1* | X | Xq22.3 | 3 | Association and other |
| *YWHAZ* | 8 | 8q23.1 | 3 | Association and other |
| *ZCCHC13* | X | Xq13.2 | 3 | Association and other |
| *RTL4* | X | Xq23 | 3 | Association and other |
| *ZNF157* | X | Xp11.2 | 3 | Association and other |

**Supp. Table S6** The criteria of ACMG-AMP guidelines used to determine variant classifications

| **Sample** | **cDNA** | **Protein** | **Genotype** | **Gene** | **Type** | **Origin** | **Inheritance Pattern** | **Classification** | **ACMG**  **classification** | **ACMG-AMP criteria** |
| --- | --- | --- | --- | --- | --- | --- | --- | --- | --- | --- |
| AU076603 | c.1229delC | p.Pro410fs | het | *IQSEC2* | fs del | de novo or father | XLD | LP | P | PVS1, PM2, PP4 |
| AU065903 | c.766C>T | p.Arg256* | het | *MEF2C* | stopgain | de novo | AD | P | P | PVS1, PS2, PM2, PP3, PP4, PP5 |
| AU049703 | c.403-1G>T | - | het | *MEF2C* | splicing | de novo | AD | P | P | PVS1, PS2, PM2, PP3, PP4 |
| AU012204 | c.973C>T | p.Arg325* | het | *MBD5* | stopgain | de novo | AD | P | P | PVS1, PS2, PM2, PP3, PP4 |
| AU060803 | c.404dupG | p.Gly136fs | het | *PTEN* | fs ins | father | AD | LP | P | PVS1, PM2, PP4, BS2 |
| AU037503 | c.460dupC | p.Arg154fs | het | *PTEN* | fs ins | mother | AD | LP | P | PVS1, PM2, PP4, BS2 |
| AU095803 | c.2854C>T | p.Arg952* | hom | *CDKL5* | stopgain | mother | XLD | LP | Likely Benign | PVS1, BS2, BP4, BP6 |
| AU048503 | c.803+1G>A | - | het | *HEPACAM* | splicing | father | AD/AR | LP | VUS | PM2, PP3, PP4, BS2 |
| AU065403 | c.1742dupT | p.Leu581fs | het | *NF1* | fs ins | mother | AD | LP | VUS | PVS1, PP4, BS2 |
| AU065503 | c.1742dupT | p.Leu581fs | het | *NF1* | fs ins | mother | AD | LP | VUS | PVS1, BS2 |
| AU099703 | c.1742dupT | p.Leu581fs | het | *NF1* | fs ins | mother | AD | LP | VUS | PVS1, BS2 |
| AU052603 | c.1015delG | p.Val339fs | het | *RNF135* | fs del | father | AD | P | VUS | PP5, BS2 |
| AU095503 | c.1015delG | p.Val339fs | het | *RNF135* | fs del | father | AD | P | VUS | PP5, BS2 |
| AU056603 | c.3424_3425del | p.Leu1142fs | het | *SHANK3* | fs del | de novo | AD | P | P | PVS1, PS2, PM2, PP4 |
| AU013503 | c.3679dupG | p.Ala1227fs | het | *SHANK3* | fs ins | de novo or father | AD | P | P | PVS1, PP4, PP5, BS1 |
| AU035703 | c.3679dupG | p.Ala1227fs | het | *SHANK3* | fs ins | de novo | AD | P | P | PVS1, PS2, PP4, PP5, BS1 |
| AU039303 | c.4753_4763del | p.Lys1585fs | het | *TSC2* | fs del | de novo | AD | P | P | PVS1, PS2, PM2, PP4 |
| AU018703 | c.199G>A | p.Asp67Asn | het | *MAP2K1* | missense | de novo or father | AD | P | P | PS1, PM1, PM2, PP2, PP3, PP4, PP5 |
| AU017403 | c.1081C>G | p.Leu361Val | het | *TSC2* | missense | mother | AD | LP | LP | PM1, PM2, PP2, PP3, PP5, BS2 |

fs del: frameshift deletion, fs ins: frameshift insertion

XLD: X-linked dominant, AD: autosomal dominant, AR: autosomal recessive

P: Pathogenic, LP: Likely pathogenic, VUS: Variants of uncertain significance

**Supp. Table S7.** GSAMD and CytoScan HD results for 15q13.3 microduplications

| **Sample** | **Position** | **Size (kbp)** | **Band** | **Type** | **Confidence** | **# Markers** |
| --- | --- | --- | --- | --- | --- | --- |
| **GSAMD** | | | | | | |
| AU096503 | 15:32067075-32442808 | 375.73 | 15q13.3 | dup | 238.38 | 88 |
| AU032303 | 15:32001237-32515100 | 513.86 | 15q13.3 | dup | 278.32 | 100 |
| AU044903 | 15:32025034-32442808 | 417.77 | 15q13.3 | dup | 326.50 | 96 |
| **CytoScan HD** | | | | | | |
| AU032303 | 15:33999631-32444261 | 444.63 | 15q13.3 | dup |  | 577 |

**Supp. Table S8.** Phenotypes of carriers with pathogenic and likely pathogenic variants of syndromic genes and recurrent CNVs

| **Sample** | **Syndrome** | **Previously Reported Clinical Characteristics** | **Clinical Symptoms of the Carrier** |
| --- | --- | --- | --- |
| AU076603 | Mental retardation, X-linked 1/78 | Mild to severe intellectual disability, and some had seizures, language delay, and nonspecific facial dysmorphism (females were much less severely affected) | Moderate intellectual disability, severe language delay (spoke her first word at 7 years old) and lack phrase speech, delayed psychomotor development, abnormal EEG with too much slow wave activity, and her father also had a language delay |
| AU065903 | Chromosome 5q14.3 deletion syndrome | Mental retardation, absent speech, seizures, poor eye contact, and stereotypic movements, some had hypotonia, delayed motor development, and variable brain anomalies on imaging | Mental retardation, febrile seizures (onset at around age 1 year), lack of speech, poor eye contact, restricted or stereotyped interests and activities, poor motor coordination and abnormal gait, delay of motor development, abnormal MRI, and sleep problems |
| AU049703 | Chromosome 5q14.3 deletion syndrome | Mental retardation, absent speech, seizures, poor eye contact, and stereotypic movements, some had hypotonia, delayed motor development, variable dysmorphic features, and variable brain anomalies on imaging | Mental retardation, epilepsy, lack of speech, poor eye contact, restricted or stereotyped interests and activities, poor motor coordination and abnormal gait, motor development delay, having sleep problems; his mother also had epilepsy, and he has a family history of schizophrenia, cerebral palsy, febrile seizure, and undiagnosed development disabilities. |
| AU012204 | Mental retardation | Mental retardation, developmental delay, motor delay, severe language impairment, and autistic-like behavioral problems, some have seizures, hypotonia, feeding difficulties, sleep disturbances, short stature, craniofacial abnormalities | Mental retardation, infantile hypotonia, global developmental delay (walked independently at age 22 months and said his first word and phrase at age 37 months), sleep disturbances, short stature (-2.8SD), congenital malformations (funnel chest, delayed closure of fontanel), abnormal imaging (scalp atrophy), normal hearing and vision, and no seizure |
| AU060803 | PTEN hamartoma-tumor syndrome | Macrocephaly | Abnormal head shape, delayed closure of fontanel, ribs valgus |
| AU037503 | PTEN hamartoma-tumor syndrome | Macrocephaly | Large head circumference during pregnancy |
| AU048503 | Megalencephalic leukoencephalopathy with subcortical cysts (recessive); leukodystrophy and macrocephaly (dominant) | Infantile-onset of macrocephaly, mildly delayed motor development, some have mild residual hypotonia or clumsiness, and mental retardation | Development delay (walked independently at age 15 months, spoke her first word at age 41 months and can only say less than 5 words), mental retardation and head circumference data is unavailable |
| AU065403 | Neurofibromatosis, type 1 | Cafe-au-lait spots, Lisch nodules in the eye, fibromatous tumors of the skin, some children have learning, behavioral, and vision problems, scoliosis, macrocephaly, short stature, pseudarthrosis, difficulties with coordination, and a mild form of epilepsy | Development delay (walked independently at age 14 months, spoke his first word at age 45 months and his first phrase at age 47 months), vision problem (amblyopia), abnormal EEG |
| AU065503 | Neurofibromatosis, type 1 | Cafe-au-lait spots, Lisch nodules in the eye, fibromatous tumors of the skin, some children have learning, behavioral, and vision problems, scoliosis, macrocephaly, short stature, pseudarthrosis, difficulties with coordination, and a mild form of epilepsy | Development delay, feeding difficulties, skeletal abnormality: leg bowing, early closure of cranial suture, skin, tooth and ear abnormalities, difficulty with balance and movement coordination, hyperactive, compulsive and aggressive behaviors, and sleep problems |
| AU099703 | Neurofibromatosis, type 1 | Cafe-au-lait spots, Lisch nodules in the eye, fibromatous tumors of the skin, some children have learning, behavioral, and vision problems, scoliosis, macrocephaly, short stature, pseudarthrosis, difficulties with coordination, and a mild form of epilepsy | Development delay (walked independently at age 16 months, spoke his first phrase at age 36 months), abnormal gait, poor movement coordination, hypotonia |
| AU052603 | Overgrowth syndrome | Increased postnatal height and weight, macrocephaly, learning disability, dysmorphic facial features | Language delay, febrile seizures, hyperactive, delayed closure of fontanel, abnormal gait, and sleep problems |
| AU095503 | Overgrowth syndrome | Increased postnatal height and weight, macrocephaly, learning disability, dysmorphic facial features | Language delay, hyperactive, and sleep problems |
| AU056603 | Chromosome 22q13.3 deletion syndrome | Severe verbal and social deficits | Mental retardation, global developmental delay (walked independently at age 18 months, spoke her first word at age 40 months, and can only say less than 5 words), severe social deficits and typical autistic behavior problems, strabismus, inverted eyelashes, late teething, poor motor coordination and abnormal gait, and sleep problems |
| AU013503 | Chromosome 22q13.3 deletion syndrome |  | Severe social deficits, spoke her first word at age 45 months and first phase at age 48 months; her father also suffered from language delay |
| AU035703 | Chromosome 22q13.3 deletion syndrome |  | Severe social deficits, spoke her first word and phase at age 24 months, but lost language skills at 42 months, abnormal gait, hyperactive, and sleep problems |
| AU039303 | Tuberous sclerosis-2 | Tuberous sclerosis, epilepsy, intellectual disturbance, and sebaceous adenoma | Tuberous sclerosis and epilepsy |
| AU018703 | Cardio-facio-cutaneous syndrome-3 | Characteristic craniofacial features, cardiac anomalies, hair and skin abnormalities, postnatal growth deficiency, hypotonia, and developmental delay | Congenital heart disease, development delay (walked independently at age 20 months), and sleep problems |
| AU017403 | Tuberous sclerosis-2 | Tuberous sclerosis, epilepsy, intellectual disturbance, and sebaceous adenoma | Abnormal MRI (abnormal left frontal lobe), masturbation syndrome |
| AU077403 | 15q11-13 duplication syndrome | Moderate to severe intellectual disability, ataxia, hypotonia, epilepsy, developmental delays, language is impaired or absent in most, no dysmorphic features, and congenital malformations are rare | A four-year-old male with autism, harboring a 15q11.2-12 duplication, has low muscle tone and his muscles feel soft. He displayed problems in motor coordination, especially gross motor movement such as walking unstably; as well as displayed developmental delay; intellectual disability; and impaired language. His standard score of the adaptive behavior composite evaluated by Vineland Adaptive Behavior Scales (VABS) is 60. |
| AU096503 |  |  | A two-year-old male with ASD; he carries the 15q13.3 duplication. He has a family history of mental retardation and showed developmental delay and is language impaired. |
| AU032303 |  |  | A four-year-old male with typical autism, harboring a 15q13.3 duplication, has a family history of developmental delay and almost no language and intellectual deficiency. VABS’s standard score is 55. In addition, he has rough skin and decreased sensitivity to pain. |
| AU044903 |  |  | A five-year-old male with autism; he also carries a 15q13.3 duplication. He has a family history of language delay and epilepsy and showed poor motor coordination and abnormal gait. He has speech and language delay and deficits, and was noted to be hyperactive. |
| AU042703 | 2q37 deletion syndrome | Mild facial dysmorphism, vascular and skeletal malformations, a variable degree of intellectual disability, and hypotonia | A three-year-old girl with autism carrying a 2q37 deletion. She also suffered from rickets, which affects the development of bones, and hernia. She has a family history of language delay and epilepsy and exhibited a general developmental delay and mental retardation. |
| AU033603 | 22q11.2 deletion syndrome | Variable symptoms | A three-year-old girl with autism has a *de novo* 22q11.2 deletion. She exhibited ear malformation, loss of hearing in the left ear, dental anomalies, strabismus, and amblyopia. She has a severe milk allergy, serious feeding problems, no language, cognitive deficit, and developmental delay (walking independently by 36 months). While lacking the symptoms of congenital heart disease and palatal abnormalities, we found many features that were previously reported and consistent with this syndrome’s phenotype. |

**Supp. Table S9.** Phenotypes of carriers of *SHANK2* and *SHANK3* variants

| **Sample ID** | **AU056603** | **AU013503** | **AU035703** | **AU067003** | **AU102003** | **AU074003** |
| --- | --- | --- | --- | --- | --- | --- |
| **Variant** | *SHANK3* c.3424_3425del (p.Leu1142fs) | *SHANK3* c.3679dupG (p.Ala1227fs) | *SHANK3* c.3679dupG (p.Ala1227fs) | *SHANK3* c.593C>G (p.Ala198Gly) | *SHANK3* c.898C>T (p.Pro300Ser) | *SHANK2* c.2540_2541del (p.Ser847*) |
| **Gender** | Female | Female | Female | Male | Male | Male |
| **Age** | 3y6m | 4y7m | 5y4m | 5y1m | 5y4m | 4y |
| **Socialization domain of VABS (mental age)** | NA | NA | 2m | 4m | 6m | 4m |
| **Motor development delay** | － | － | － | － | － | － |
| **Language development delay** | ＋ | ＋ | ＋ | ＋ | ＋ | ＋ |
| **Unusual sensory interests** | － | － | ＋ | － | － | ＋ |
| **Hyper-responsivity to sensory stimuli** | － | － | － | ＋ | － | ＋ |
| **Macrocephaly** | － | NA | － | － | － | － |
| **Microcephaly** | － | NA | － | － | － | － |
| **Abnormal EEG** | NA | － | NA | NA | NA | － |
| **Abnormal MRI** | NA | － | － | NA | NA | NA |
| **Skills loss** | － | － | ＋ | － | － | － |
| **Epilepsy** | － | － | － | － | － | － |
| **Sleep disorder** | ＋ | － | ＋ | ＋ | ＋ | ＋ |
| **Gastrointestinal problems** | ＋ | － | ＋ | － | － | － |
| **Hypotonia** | － | － | － | － | － | － |
| **Hypertonia** | － | － | － | － | － | － |
| **Hyperactivity** | － | － | ＋ | ＋ | ＋ | － |
| **Anxiety** | － | － | － | － | － | － |
| **Aggressive behavior** | － | － | ＋ | － | － | － |
| **Obsessive behavior** | － | － | － | － | ＋ | － |
| **Feeding difficulty** | － | － | － | － | － | － |
| **Abnormal gait** | ＋ | － | ＋ | ＋ | ＋ | － |
| **Other problems** | Inverted eyelashes, strabismus | Decayed tooth, myopia and astigmatism | － | － | － | － |

**Supp. Table S10.** Phenotypes of carriers of 15q11-13 duplications

| **Sample ID** | **AU077403** | **AU096503** | **AU032303** | **AU044903** |
| --- | --- | --- | --- | --- |
| **Variant** | 15q11.2-12 | 15q13.3 | 15q13.3 | 15q13.3 |
| **Gender** | Male | Male | Male | Male |
| **Age** | 4y5m | 2y9m | 4y6m | 5y2m |
| **Mental retardation** | ＋ | NA | ＋ | NA |
| **Socialization domain of VABS (mental age)** | 4m | NA | 2m | NA |
| **Motor development delay** | ＋ | － | － | － |
| **Language development delay** | ＋ | ＋ | ＋ | ＋ |
| **Unusual sensory interests** | ＋ | － | ＋ | ＋ |
| **Hyper-responsivity to sensory stimuli** | ＋ | － | － | ＋ |
| **Macrocephaly** | － | － | － | － |
| **Microcephaly** | － | － | － | － |
| **Abnormal EEG** | － | － | NA | NA |
| **Abnormal MRI** | － | － | NA | NA |
| **Skills loss** | － | － | － | － |
| **Epilepsy** | － | － | － | － |
| **Sleep disorder** | － | － | － | － |
| **Gastrointestinal problems** | ＋ | － | － | － |
| **Hypotonia** | ＋ | － | － | － |
| **Hypertonia** | － | － | － | － |
| **Hyperactivity** | － | － | ＋ | ＋ |
| **Anxiety** | － | － | － | － |
| **Aggressive behavior** | － | ＋ | ＋ | － |
| **Obsessive behavior** | － | － | ＋ | ＋ |
| **Feeding difficulty** | － | － | － | － |
| **Abnormal gait** | － | － | － | ＋ |
| **Poor coordination of movements** | ＋ | － | － | ＋ |
| **Other problems** | Suspected skeletal abnormality | － | Rough skin | Suspected joint abnormality |

**Supp. Table S11.** Comparison of variants between cases with and without a specific phenotype

| **Phenotype** | **# Cases with specific phenotype** | **# Cases without specific phenotype** | **Unadjusted *p* value** | **Adjusted *p* value** |
| --- | --- | --- | --- | --- |
| **Loss of language skills** | 43 | 471 | 0.091 | 0.73 |
| **Minimally verbal** | 113 | 330 | 0.093 | 0.75 |
| **Unusual sensory interests** | 49 | 205 | 0.22 | 1 |
| **Self-injurious behavior** | 13 | 449 | 0.34 | 1 |
| **Epilepsy/Tics** | 31 | 432 | **0.017** | **0.13** |
| **Gastrointestinal problems** | 76 | 386 | 0.36 | 1 |
| **Hypotonia** | 22 | 420 | 0.67 | 1 |
| **Insensitivity to pain** | 120 | 304 | 0.27 | 1 |
